# Can artificial intelligence improve health performance? The mediating role of health innovation and the moderating role of digital infrastructure

**DOI:** 10.3389/fpubh.2026.1889171

**Published:** 2026-08-04

**Authors:** Anis Omri, Fadhila Hamza

**Affiliations:** 1 Department of Business Administration, College of Business and Economics, Qassim University, Buraydah, Saudi Arabia; 2 Department of Economics, Faculty of Economics and Management of Nabeul, University of Carthage, Carthage, Tunisia; 3 Department of Accounting, College of Business Administration, Princess Nourah Bint Abdulrahman University, Riyadh, Saudi Arabia

**Keywords:** artificial intelligence, developing countries, digital infrastructure, health innovation, health performance

## Abstract

This study examines whether artificial intelligence improves health performance in developing countries and identifies the mechanisms and conditions through which this effect occurs. Using panel data for 19 developing countries over the period 2015–2023, the study investigates the mediating role of health innovation and the moderating role of digital infrastructure in the relationship between artificial intelligence and health performance. The study applies Hayes’ PROCESS macro to estimate direct, mediation, moderation, and moderated mediation effects. To strengthen the reliability of the findings, two robustness checks are conducted: two-step System GMM estimation and alternative measurement using infant mortality and disaggregated artificial intelligence indicators. The results show that artificial intelligence has a positive and statistically significant effect on both life expectancy and UHC service coverage. Health innovation partially mediates these relationships, indicating that artificial intelligence improves health performance not only directly through healthcare delivery, diagnosis, surveillance, and resource allocation, but also indirectly by stimulating medical and life-science innovation. The findings further reveal that digital infrastructure strengthens artificial intelligence’s effect on health innovation and amplifies its direct effect on both health performance indicators. The moderated mediation results confirm that the artificial intelligence–health innovation–health performance pathway strengthens as digital infrastructure improves. The robustness tests support these conclusions. System GMM confirms the partial mediation mechanism after accounting for dynamic panel-data concerns, while the alternative-measure analysis shows that artificial intelligence patent applications, industrial robots, and artificial intelligence scholarly publications reduce infant mortality through health innovation under stronger digital infrastructure. These findings indicate that artificial intelligence yields greater health benefits when combined with health innovation capacity and digital infrastructure, while also showing that not all artificial intelligence dimensions contribute equally to health-system improvement.

## 1 Introduction

Health performance has become a central concern in public health and development research because high-quality health systems are essential for improving survival, protecting populations from preventable disease, and advancing equitable human development (1, 2). This concern is especially important in developing countries, where health systems frequently operate under resource constraints, fragmented service delivery, uneven access to care, shortages of trained health workers, and persistent gaps in primary health care capacity (3, 4). The COVID-19 pandemic further exposed the vulnerability of health systems that lack strong information systems, real-time surveillance capacity, digital coordination mechanisms, and innovation-based preparedness, thereby reinforcing the need for health systems that are not only accessible but also resilient, adaptive, and technologically capable (5, 6). Recent public health literature increasingly emphasizes that improving health outcomes requires more than expanding physical health infrastructure; it also requires better use of data, innovation, digital technologies, and decision-support systems that can improve prevention, diagnosis, monitoring, and resource allocation (1, 7). These requirements are highly relevant to developing countries because the burden of under-resourced health systems is often compounded by rapid population growth, epidemiological transition, and rising demand for affordable, high-quality care (4). Accordingly, examining the technological determinants of health performance has become an important research priority, particularly in countries seeking to improve universal health coverage, strengthen service delivery, and enhance long-term population health under limited fiscal and institutional capacity.

Artificial Intelligence (AI) has emerged as one of the most important digital technologies with the potential to reshape health systems and public health practice through improved diagnosis, disease prediction, clinical decision support, medical imaging, patient monitoring, public health surveillance, and health-service management (7, 8). In healthcare delivery, AI can support patient administration, clinical decision-making, patient monitoring, and healthcare interventions, thereby creating opportunities to reduce inefficiencies and improve the responsiveness of health services (9). In public health, AI can strengthen disease surveillance, emergency response, health communication, and population-level risk prediction by processing large and complex datasets more rapidly than conventional methods (5, 6). These capabilities are particularly relevant for developing countries because AI may help compensate for shortages of skilled health workers, improve screening and follow-up, support primary health care, and extend health services to underserved populations when implemented responsibly (3, 4). However, the health benefits of AI should not be assumed to occur automatically, because AI can also reinforce inequalities, produce biased outputs, create governance challenges, and fail to generate population-level benefits when health systems lack adequate infrastructure, regulation, and implementation capacity (2). Recent evidence therefore suggests that AI in health should be understood as a socio-technical transformation rather than a purely technical upgrade, because its contribution depends on the ability of countries to connect algorithms, data systems, institutions, professionals, and innovation ecosystems in ways that serve public health objectives (5). This is particularly important in developing economies, where AI adoption remains uneven and where weak digital infrastructure, limited data quality, fragmented health information systems, and insufficient innovation capacity may prevent AI capability from being translated into better health performance (3). Thus, a macro-level study of AI and health performance should move beyond the direct association between AI and health outcomes and examine the mechanisms through which AI generates health-system improvements and the contextual conditions that make these mechanisms more effective.

Health innovation represents a central mechanism through which AI can influence health performance because AI can accelerate the production, diffusion, and application of medical knowledge and health-related technologies (7, 8). AI enables researchers, firms, and health institutions to analyze biomedical data, improve medical imaging, identify disease patterns, assist drug discovery, support diagnostic tools, optimize clinical workflows, and develop predictive technologies that may later be converted into health-system applications (7, 10). In this study, health innovation is captured through life-science patents granted by technology, particularly medical technology patents, because patent indicators provide an observable and internationally comparable measure of codified inventive activity in health-related technological domains (11). Medical technology patents are especially appropriate for this research because they reflect innovation in diagnostic devices, therapeutic equipment, monitoring tools, imaging systems, medical instruments, and other technologies that can improve service quality, expand diagnostic capacity, and support more efficient health delivery (12). The use of patent-based indicators is consistent with innovation economics, where patents are widely used to measure inventive output, technological accumulation, and the direction of knowledge creation across countries and sectors (11). In the present framework, AI is expected to stimulate health innovation because AI-related capabilities improve knowledge processing, reduce research complexity, enhance experimentation, and support the development of new medical technologies (7, 8). Health innovation, in turn, is expected to improve health performance by expanding the technological base of health systems, improving diagnostic and treatment capacities, supporting earlier detection and prevention, and increasing the efficiency and quality of health-service delivery (1, 10). Accordingly, health innovation can be conceptualized as a mediating pathway through which national AI capability is converted into improved health-system coverage and population health outcomes (7, 13).

Nevertheless, the ability of AI to stimulate health innovation is likely to depend on the level of digital infrastructure available within a country (14). AI is highly dependent on digital connectivity, data availability, broadband networks, cloud systems, interoperable information systems, secure digital platforms, and widespread internet access because these conditions enable the collection, exchange, processing, and application of health-related data (2). Digital infrastructure therefore represents an enabling condition that determines whether AI capability can be transformed into practical health innovation (14). Countries with stronger internet penetration, broadband subscriptions, mobile connectivity, secure servers, and ICT capacity are more likely to support digital research networks, health-data exchange, telemedicine, electronic health records, and the development and diffusion of AI-enabled medical technologies (4). By contrast, weak digital infrastructure may reduce the innovation-enhancing effect of AI because researchers, firms, and health institutions may lack the connectivity, data systems, and digital platforms needed to develop, test, commercialize, and implement AI-enabled health technologies (2, 3). This moderating logic is particularly relevant for developing countries because the effectiveness of AI-driven health solutions often depends on whether national systems possess the foundational digital conditions needed to support primary health care, surveillance, service integration, and digital inclusion. Recent digital health research also emphasizes that digital transformation in healthcare requires systemic changes in infrastructure, workforce preparation, technology acceptance, and organizational processes, indicating that digital infrastructure is not simply a background condition but a core determinant of whether digital technologies can generate innovation and health-system benefits (14). Thus, digital infrastructure can be understood as a boundary condition that strengthens AI’s effect on health innovation and, through this channel, reinforces AI’s indirect contribution to health performance.

Despite the growing interest in AI and health, several gaps remain in the existing literature. First, much of the current research examines AI at the clinical or application level, focusing on diagnostic algorithms, clinical decision-support systems, medical imaging, disease surveillance, and digital health tools, while relatively little is known about whether AI contributes to health performance at the national level (5, 7, 8). This gap is important because the success of AI in specific clinical applications does not necessarily translate into system-wide improvements in life expectancy, universal health coverage, or population health unless AI capabilities are embedded in broader health systems, innovation structures, and public institutions. Second, previous studies often treat AI as a direct technological solution but pay less attention to the mechanisms by which AI may generate measurable health-system benefits. In particular, health innovation remains underexplored as a transmission channel, even though medical technologies, life-science patents, diagnostic tools, and biomedical innovations represent plausible pathways through which AI capabilities can be converted into better health outcomes. Third, the conditional nature of the AI–health innovation–health performance relationship remains insufficiently examined, especially in developing countries where digital infrastructure varies substantially and may determine whether AI can be transformed into practical health innovation. Fourth, although recent studies have highlighted ethical, governance, and equity concerns surrounding AI in health, less empirical attention has been paid to the structural conditions that enable AI to deliver inclusive and system-wide public health benefits. Addressing these gaps is important because AI capability alone may not improve health performance unless it is supported by health innovation capacity and the digital infrastructure required for data exchange, technological diffusion, and service integration. Against this background, this study investigates whether AI improves health performance in 19 developing countries over the period 2015–2023 by examining health innovation as a mediating mechanism and digital infrastructure as a moderating condition. Methodologically, the study applies Hayes’ PROCESS macro to estimate direct, mediation, moderation, and moderated mediation effects, and further validates the findings using two-step System GMM and alternative measurement tests based on infant mortality and disaggregated AI indicators.

This study contributes to the literature in several ways. First, it extends the digital public health literature by moving beyond clinical and application-level discussions of AI toward a macro-level explanation of how national AI capability affects health performance in developing countries. Second, it contributes to the health innovation literature by identifying health innovation as a mediating mechanism by which AI can translate into improvements in life expectancy and UHC service coverage. This contribution is important because it shows that AI improves health performance not only through direct healthcare delivery channels, such as diagnosis, surveillance, patient monitoring, and resource allocation, but also through the production of health-related innovation. Third, the study advances the digital health and development literature by introducing digital infrastructure as a boundary condition that strengthens both the AI–health innovation pathway and the direct AI–health performance relationship. This highlights that the health benefits of AI depend on digital readiness rather than AI capability alone. Fourth, the study contributes methodologically by applying a moderated mediation framework to panel data and complementing Hayes’ PROCESS analysis with two-step System GMM, which addresses concerns related to dynamic persistence, unobserved heterogeneity, reverse causality, and potential endogeneity. Fifth, the study adds measurement-based robustness evidence by showing that the main moderated mediation logic remains valid when health performance is remeasured using infant mortality, and AI is disaggregated into AI patent applications, industrial robots, AI scholarly publications, and VC investments in AI. This analysis shows that not all AI dimensions contribute equally to health-system improvement: knowledge-based and technology-based AI indicators support the conditional indirect pathway, whereas VC investments in AI do not. Finally, the study offers policy-relevant evidence for developing countries by showing that AI strategies should be integrated with health innovation policies and digital infrastructure investment if governments aim to expand service coverage, improve population health, reduce infant mortality, and strengthen health-system resilience.

The rest of the article is structured as follows: Section 2 presents the literature review and develops the hypotheses; Section 3 describes the data and empirical methodology; Section 4 presents and discusses the obtained results; and Section 5 concludes.

## 2 Literature review and hypothesis development

### 2.1 AI and health performance

AI has increasingly been recognized as a transformative force in healthcare and public health because it enhances health systems’ ability to diagnose disease, predict risks, allocate resources, personalize treatment, and improve service delivery (15, 16). In the healthcare context, AI refers to computational systems capable of learning from large datasets, identifying complex patterns, and supporting decision-making processes that traditionally depended mainly on human expertise (17). This technological capacity is particularly relevant to health performance because improved diagnostic precision, faster disease detection, streamlined clinical workflows, and more efficient use of scarce resources can directly contribute to stronger health service coverage and better population health outcomes (18). Al Kuwaiti et al. (15) show that AI applications are increasingly used across medical diagnosis, imaging, clinical decision support, patient monitoring, robotic surgery, drug discovery, and hospital management, suggesting that AI can improve the quality, speed, and responsiveness of healthcare systems. Similarly, Olawade et al. (16) argue that AI can enhance public health by improving disease surveillance, health-risk prediction, outbreak detection, healthcare planning, and preventive interventions. These functions are important because health performance is determined not only by the availability of hospitals or health expenditure but also by the capacity of health systems to identify health needs early, respond efficiently, and deliver appropriate services to the population (19). In this regard, AI can improve health outcomes through both clinical and public health channels. At the clinical level, AI supports diagnosis and treatment decisions by reducing human error, accelerating medical image interpretation, and enabling more targeted care (20). At the public-health level, AI supports surveillance, epidemic monitoring, environmental health assessment, and population-level risk management, thereby improving the ability of governments and health institutions to protect population health (21). Fisher and Rosella (19) further emphasize that public health organizations can benefit from AI when its adoption is aligned with organizational priorities, ethical governance, data readiness, and implementation capacity. This suggests that AI can strengthen health performance not merely as a technical tool, but as a strategic capability that improves prevention, planning, and decision-making in health systems (22).

The positive association between AI and health performance is also supported by studies that emphasize the economic, organizational, and efficiency gains of AI in healthcare (23, 24). Wolff et al. (24), in a systematic review of the economic impact of AI in healthcare, show that AI has the potential to reduce costs, improve efficiency, and support the better allocation of medical resources, although its economic value depends on the implementation context and the quality of evaluation. Voets et al. (18) similarly review health economic evaluations of AI in healthcare and argue that AI can offer value by improving diagnostic accuracy, reducing unnecessary procedures, and supporting more efficient clinical pathways. Khanna et al. (20) further distinguish between AI applications in diagnosis and treatment, showing that AI can generate economic and clinical benefits by improving diagnostic performance, reducing delays, and supporting more effective therapeutic decisions. Uchegbu et al. (25) also note that health economic evaluation is essential for understanding the value of AI technologies, particularly because AI can influence both costs and outcomes through improved accuracy, workflow efficiency, and clinical decision-making. These economic and operational benefits are closely related to health performance because countries with more efficient health systems can expand service coverage, improve quality, and use limited resources more effectively. At the organizational level, Li et al. (23) provide empirical evidence from a healthcare institution in the United Arab Emirates showing that AI can improve human resource performance by supporting work efficiency, decision-making, and service quality. This finding is relevant at the macro-health level because human resource performance is a core component of healthcare delivery, especially in systems facing shortages of trained professionals. Beyond healthcare institutions, AI can also improve health performance through environmental and public-safety channels. Adefemi et al. (26) show that AI can support environmental health and public safety strategies by improving monitoring, risk prediction, and emergency preparedness, which are important for preventing disease exposure and protecting population health. More recently, Ageli (27) links AI to life expectancy within a broader macroeconomic framework, suggesting that AI may contribute to population health outcomes when combined with economic and environmental factors. This evidence is directly relevant to the present study because life expectancy is one of the health performance indicators used to capture the long-term outcomes of health-system and socioeconomic improvements. Taken together, the reviewed studies indicate that AI can enhance health performance through multiple mutually reinforcing mechanisms, including improved diagnosis and treatment, stronger public health surveillance, greater healthcare efficiency, better health workforce performance, improved environmental health monitoring, and longer life expectancy. Accordingly, the following hypothesis is proposed:

*H*1: AI has a positive effect on health performance.

### 2.2 The mediating effect of health innovation

AI is increasingly conceptualized as a major driver of healthcare innovation because it enables new forms of diagnosis, personalized treatment, telehealth delivery, biomedical discovery, and data-driven clinical decision-making (28, 29). Unlike conventional digital tools, AI can process large, complex datasets, detect hidden patterns, learn from prior cases, and support the development of novel health technologies. This makes AI particularly important for health innovation, as it can accelerate the creation of medical devices, diagnostic algorithms, personalized healthcare solutions, telemedicine platforms, and biomedical research applications. Arora (28) argues that AI should be understood as a digital healthcare innovation because it changes how healthcare technologies are designed, implemented, and used in clinical settings. Similarly, da Silva (29) highlights that AI has become increasingly relevant for biomedical research and health innovation, especially in emerging economies where digital tools may help overcome limitations in research capacity and healthcare access. In personalized healthcare, Li et al. (30) show that AI supports innovation by improving disease prediction, treatment selection, patient monitoring, and individualized medical decision-making. These arguments suggest that AI can act as an upstream technological capability that stimulates health innovation by improving knowledge generation, data analytics, and the development of medical technologies.

Recent studies also show that AI-driven innovation extends beyond clinical diagnosis to include telehealth, healthcare management, and organizational transformation (31, 32). In telehealth, AI supports innovation by enabling remote diagnosis, virtual consultation, automated triage, patient follow-up, and intelligent health monitoring, thereby expanding the scope and efficiency of healthcare delivery. Amjad et al. (31) emphasize that AI-enabled telehealth innovation can improve accessibility, reduce service delays, and strengthen patient-centered care. However, AI-for-health innovation is not purely technical; it also depends on governance, infrastructure, implementation capacity, and ethical design. Farlow et al. (32) argue that global digital health and AI-for-health innovation face important challenges related to equity, scalability, data governance, and contextual adaptation. This is particularly important for developing countries, where AI may generate health innovation only when digital and institutional conditions allow new technologies to be developed, tested, and integrated into health systems. Neher et al. (33) further show that healthcare leaders perceive AI as an innovation with distinctive characteristics, including complexity, adaptability, and potential organizational impact. This means that AI can foster health innovation not only by generating new technologies, but also by changing healthcare routines, improving decision processes, and supporting new models of care. Patil (34) similarly views AI as a mechanism for promoting innovation across sensor-based, IoT-enabled, and health-science applications. Therefore, the first link in the mediating chain is theoretically justified: AI contributes positively to health innovation.

Health innovation is also expected to improve health performance because innovative technologies, processes, and care models can enhance service quality, organizational efficiency, patient outcomes, and population health (35, 36). Moreira et al. (36) show that innovation can influence the performance of healthcare organizations by improving operational processes, service delivery, and organizational responsiveness. Madden et al. (35), in a systematic review, confirm that healthcare innovation can affect measurable organizational outcomes, including efficiency, quality, safety, patient experience, and clinical performance. In Saudi Arabia, Akinwale and AboAlsamh (37) provide empirical evidence that technological innovation improves healthcare performance, supporting the argument that innovation can strengthen health-service delivery in developing and emerging contexts. Hussain et al. (38) also link sustainable healthcare innovation to improvements in health economics, social policy, and management, suggesting that innovation contributes to broader system performance rather than only isolated clinical outcomes. At the population-health level, Van Nuys et al. (39) show that innovation in heart failure treatment can improve life expectancy and reduce disability, while Muradov et al. (40) demonstrate that medical innovation matters for life expectancy when combined with health expenditure and economic complexity. Taken together, these studies suggest that AI improves health performance indirectly by stimulating health innovation, which then enhances healthcare quality, service coverage, efficiency, and longevity. Accordingly, the following hypothesis is proposed:

*H*2: Health innovation mediates the relationship between AI and health performance.

### 2.3 The moderating effect of digital infrastructure

Digital infrastructure can strengthen the relationship between AI and health innovation because AI requires a supportive digital environment to generate, diffuse, and implement new healthcare solutions (41, 42). Although AI can improve data processing, predictive analytics, diagnosis, telehealth, and decision-making, these capabilities are unlikely to produce strong health innovation when countries lack reliable digital connectivity, interoperable data systems, broadband networks, cloud-based platforms, secure servers, and digitally enabled healthcare processes. Swan et al. (42) argue that AI in healthcare creates value through interaction with broader digital health systems, suggesting that AI’s contribution depends on complementary digital foundations that allow patients, providers, and organizations to co-create value. Similarly, Feroz and Kwak (41) emphasize that AI and digital technologies increasingly converge in organizations, where digital platforms, data systems, and technology-enabled processes provide the foundation for intelligent innovation. This logic is consistent with Reier Forradellas and Garay Gallastegui (43), who show that AI and digital technologies jointly reshape economic structures, regulatory environments, and innovation opportunities. In the health context, digital infrastructure supports AI-driven innovation by enabling electronic records, telemedicine, digital monitoring, cloud-based data exchange, connected health devices, and interoperable health-information systems, all of which are necessary for transforming AI capabilities into medical technologies and service innovations. Akinola and Telukdarie (44) demonstrate that digitally enabled healthcare infrastructure can advance vascular health innovation, indicating that health innovation depends not only on AI tools but also on the broader digital systems into which these tools are embedded. Koebe (45) further shows that digital technologies and AI can contribute to health-related SDGs, reinforcing the idea that their combined use can improve health access, treatment effectiveness, and system-level outcomes. In addition, Oulefki et al. (46) highlight that digital twins and AI are transforming healthcare systems through data-driven decision-making and innovation, which supports the argument that advanced digital infrastructure enhances the innovation value of AI. More broadly, Dvouletý et al. (47) argue that AI, digitalization, and new technologies are jointly reshaping research and management, suggesting that AI’s innovative impact is strongest when embedded in wider digital systems. Recent evidence from sustainable development research also confirms this moderating logic by showing that the digital economy can shape the impact of AI on broader performance outcomes, implying that AI does not operate independently from the digital context in which it is deployed (48).

Beyond strengthening the AI–health innovation pathway, digital infrastructure may also moderate the direct relationship between AI and health performance. This is because AI can influence health outcomes not only through medical technology patents and other innovation outputs, but also through more immediate improvements in healthcare delivery, public health surveillance, patient monitoring, remote consultation, diagnostic support, administrative efficiency, and data-driven resource allocation. However, these direct health-system benefits depend strongly on whether countries possess the digital foundations needed to implement AI at scale. In countries with advanced digital infrastructure, AI tools can be more effectively integrated into electronic health records, telemedicine platforms, hospital information systems, disease-surveillance networks, digital diagnostic systems, and patient-management applications. Under such conditions, AI is more likely to improve health-service coverage, accelerate medical decision-making, reduce service delays, support early detection, and enhance the efficiency of healthcare delivery. By contrast, when digital infrastructure is weak, AI capability may remain concentrated in research institutions, private providers, or isolated pilot projects, limiting its ability to produce system-wide health-performance gains. Therefore, digital infrastructure should be viewed not only as an enabler of AI-driven health innovation, but also as a condition that determines whether AI can directly improve health performance. Accordingly, in this study, digital infrastructure is expected to function as a dual boundary condition: it strengthens the influence of AI on health innovation and also amplifies the direct impact of AI on health performance. When digital infrastructure is high, AI is more likely to stimulate medical technology patents, digital health tools, and innovative healthcare solutions, while also directly improving universal health coverage and life expectancy through better health-system delivery. When digital infrastructure is weak, both the innovation-enhancing and direct health-performance effects of AI are likely to be constrained.

Consequently, the following hypotheses are proposed:

*H*3: Digital infrastructure positively moderates the relationship between AI and health innovation, such that the effect of AI on health innovation becomes stronger when digital infrastructure is higher.

*H*4: Digital infrastructure positively moderates the relationship between AI and health performance, such that the effect of AI on health performance becomes stronger when digital infrastructure is higher.

### 2.4 The moderated mediation effect

The preceding arguments suggest that the relationship between AI, health innovation, and health performance should be understood as a conditional indirect process rather than a simple mediation pathway (13, 49). AI is expected to improve health performance partly by stimulating health innovation, because AI enhances biomedical research, diagnostic development, personalized healthcare, telehealth solutions, and medical technology invention (28–30). In turn, health innovation improves health performance by enhancing healthcare quality, organizational efficiency, clinical outcomes, service delivery, and population-level health outcomes (35, 36). However, the strength of this indirect impact is likely to depend on the level of digital infrastructure, because AI-driven health innovation requires digital connectivity, interoperable systems, data-sharing capacity, digital platforms, and technology-enabled organizational processes (41, 42). When digital infrastructure is strong, AI can be more effectively transformed into medical technology patents, digital health applications, telehealth systems, data-driven decision tools, and other forms of health innovation that subsequently improve health performance (44, 46). Conversely, when digital infrastructure is weak, the innovation-enhancing effect of AI may be constrained because health systems, firms, and research institutions may lack the digital foundations required to develop, test, diffuse, and apply AI-enabled health technologies (32, 45). This means that the indirect impact of AI on health performance through health innovation is not constant across countries; rather, it becomes stronger in contexts where digital infrastructure supports the conversion of AI capabilities into health-related innovation and practical health-system improvements. Accordingly, digital infrastructure is expected to condition the first stage of the mediation pathway, strengthening the effect of AI on health innovation and thereby amplifying the indirect contribution of AI to health performance (13). Therefore, the study proposes the following hypothesis:

*H*5: The indirect effect of AI on health performance through health innovation is stronger when digital infrastructure is higher.

**Figure 1:**
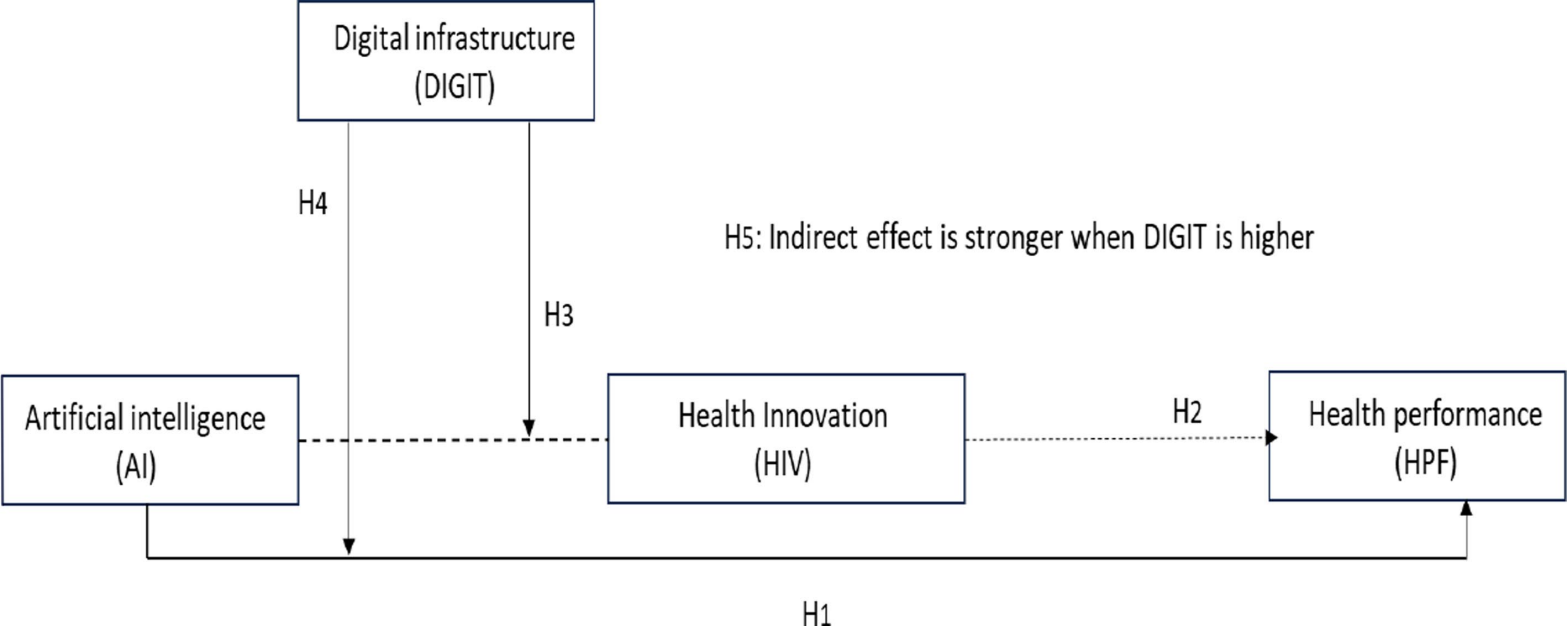
Conceptual model.

In this context, Figure 1 illustrates the study’s research model, depicting AI as the key explanatory variable, health innovation as the mediating mechanism, and digital infrastructure as the moderating factor that conditions both the AI–health innovation pathway and the direct AI–health performance relationship. The model therefore reflects a conditional process framework in which AI is expected to improve health performance directly and indirectly through health innovation, while both effects are expected to become stronger when the level of digital infrastructure is higher.

## 3 Data and methodology

### 3.1 Variable measurement and data sources

This study uses a panel dataset covering 19 developing countries^1^ over the period 2015–2023 to examine how AI affects health performance through health innovation and under different levels of digital infrastructure. The variables are selected to reflect the main components of the proposed conditional process framework: AI as the principal explanatory variable, health performance as the outcome variable, health innovation as the mediating mechanism, and digital infrastructure as the moderating condition. To ensure consistency and comparability across countries and years, the data are obtained from internationally recognized databases, including Our World in Data, the International Federation of Robotics, the World Development Indicators, the United Nations E-Government Survey, and the (64). Where composite indicators are required, the study applies arithmetic mean imputation for missing observations, min–max standardization to place variables on a comparable scale, and principal component analysis to construct synthetic indices that capture the multidimensional nature of AI and digital infrastructure.

The dependent variable is health performance, assessed using two alternative indicators. The first indicator is life expectancy at birth, expressed as the total number of years a newborn is expected to live if current mortality patterns remain unchanged. It captures overall population health and reflects long-term progress in health conditions, disease prevention, standards of living, and the effectiveness of healthcare systems. The second indicator is the UHC service coverage index, which assesses the extent to which essential health services are available across key areas, including reproductive, maternal, newborn, and child health; infectious diseases; noncommunicable diseases; and service capacity and access. This index ranges from 0 to 100, with higher scores indicating broader coverage of essential health services. Both indicators are obtained from the World Development Indicators.

Building on this health-performance framework, the independent variable in this study is AI, measured using a composite AI Index, which captures the multidimensional nature of national AI capability by combining four indicators: annual patent applications related to AI, the number of industrial robots, annual scholarly publications on AI, and venture capital investments in AI in USD millions. These indicators reflect AI-related invention, diffusion of automation, scientific knowledge production, and financial investment in AI activities. The AI-related patent applications, scholarly publications, and venture capital investment indicators are obtained from Our World in Data, while the number of industrial robots is obtained from the International Federation of Robotics. To construct the AI index, missing values are first replaced using the arithmetic mean. The indicators are then standardized using the min–max method and aggregated through principal component analysis. The PCA results for the AI index are reported in Appendix A1. The results show that two principal components have eigenvalues greater than one and jointly explain 93.06% of the total variance. The KMO statistic is 0.5715, and Bartlett’s test is statistically significant (χ^2^ = 579.0091, *p* < 0.001), confirming that the selected indicators are suitable for PCA. Higher values of the AI index indicate stronger national AI capability. Accordingly, AI is expected to positively affect health performance because stronger AI capabilities can improve diagnosis, disease prediction, clinical decision-making, public health surveillance, resource allocation, and the efficiency of healthcare delivery (15, 16). To make the construction of the AI Index more transparent, the final composite measure was obtained in four steps. First, missing values were imputed using the arithmetic mean to preserve the balanced country-year structure. Second, the four AI indicators were normalized using min-max standardization so that higher values consistently reflected stronger AI capability. Third, PCA was applied to the standardized indicators, and the retained components were selected according to the eigenvalue-greater-than-one rule. Fourth, the retained component scores were combined using eigenvalue-based weights, calculated as each retained component’s eigenvalue divided by the sum of all retained components’ eigenvalues, and the resulting score was rescaled to range from 0 to 1. Appendix Tables A1 and A2 report the PCA loadings, retained-component weights, and the correlation matrix used to construct the AI Index.

Extending this logic to the transmission mechanism, the mediating variable in this study is health innovation. Previous studies suggest that health innovation can be captured through several input-based indicators, including gross domestic R&D expenditure on health as a percentage of GDP and the number of health researchers per million inhabitants (50–52). Gross domestic R&D expenditure on health reflects the financial resources devoted to health-related research and development, while health researchers per million inhabitants capture the availability of specialized human capital involved in health research and innovation. These indicators are useful because they measure the knowledge and resource inputs that support healthcare innovation and technological advancement. However, data for these indicators were not available from the (63) for most of the countries included in our sample. Therefore, to ensure broader country coverage and maintain a balanced empirical framework, this study follows Cinaroglu and Baser (53) and measures health innovation using life-science patents granted by the filing office, which capture patents granted in the fields of medical technology, biotechnology, and pharmaceuticals. This indicator reflects health-related inventive activity in areas such as medical devices, diagnostic tools, pharmaceutical products, biotechnological processes, genetic engineering techniques, and other life-science technologies. It is particularly appropriate for the present study because health innovation is the mechanism by which AI capabilities are transformed into practical technologies that improve diagnosis, treatment, prevention, and healthcare delivery. The data are obtained from Our World in Data, based on the (64). Higher values indicate stronger life-science innovation output. Since the indicator is measured by filing office, it reflects patents granted at a given patent office rather than only patents invented by residents of the country. This limitation is acknowledged in the study, but the measure remains suitable because it provides an internationally comparable proxy for health-related inventive activity across the sampled countries. In this framework, health innovation is expected to mediate the AI–health performance relationship, as AI can spur the development of new health technologies, which in turn can improve healthcare quality, service efficiency, and population health outcomes (28, 35).

Complementing the mediating pathway, the study also uses the Digital Infrastructure Index (DIGIT) as a first-stage moderating variable. This index captures the extent to which countries possess the digital foundations required to support AI deployment, data exchange, telemedicine, digital health platforms, and health innovation. The DIGIT index is constructed from 10 indicators that reflect digital infrastructure, digital service delivery, digital participation, Internet adoption, and ICT-related trade. These indicators include individuals using the Internet, mobile cellular subscriptions, fixed broadband subscriptions, fixed telephone subscriptions, the Telecommunication Infrastructure Index, the Online Service Index, the E-Participation Index, ICT goods exports, ICT goods imports, and ICT services exports. The Telecommunication Infrastructure Index, Online Service Index, and E-Participation Index are obtained from the United Nations E-Government Survey, while internet use, broadband subscriptions, mobile and fixed telephone subscriptions, and ICT trade indicators are obtained from the World Development Indicators. As with the AI index, missing observations are replaced with the arithmetic mean, the indicators are standardized using the min–max method, and PCA is then applied to construct the composite index. The PCA results for DIGIT are reported in Appendix A2. The results indicate that four principal components have eigenvalues greater than one and jointly explain 79.51% of the total variance. The KMO statistic is 0.5724, and Bartlett’s test is statistically significant (χ^2^ = 1194.8959, *p* < 0.001), confirming that the 10 indicators are sufficiently correlated for PCA. Higher DIGIT values indicate stronger national digital infrastructure. Therefore, digital infrastructure is expected to strengthen the effect of AI on health innovation and health performance because advanced digital connectivity, broadband capacity, e-government readiness, and ICT systems facilitate the deployment, diffusion, and practical use of AI-enabled healthcare solutions (42, 46). The construction of the Digital Infrastructure Index follows the same transparent procedure. The 10 indicators were first mean-imputed where needed and then min-max standardized. PCA was then applied to the standardized indicators, and the four components with eigenvalues greater than one were retained. The final DIGIT score was calculated as an eigenvalue-weighted combination of the retained component scores and was rescaled between 0 and 1. Appendix Tables A3 and A4 provide the PCA loadings, retained-component weights, and correlation matrix for the Digital Infrastructure Index. Although the KMO statistic is slightly below the conventional 0.60 threshold, Bartlett’s test is statistically significant, indicating that the indicators are sufficiently correlated for dimensionality reduction. Accordingly, the DIGIT Index is interpreted as a broad measure of national digital readiness and is used with appropriate caution.

The model includes three control variables that may independently affect health performance and are therefore included to isolate the specific effects of AI, health innovation, and digital infrastructure. First, GDP per capita is measured as GDP per capita in constant 2015 US dollars and is obtained from the World Development Indicators. It captures the level of economic development and reflects a country’s capacity to improve living standards, expand infrastructure, and finance health-enhancing services. Second, domestic general government health expenditure is measured as the share of general government expenditure allocated to health and is also obtained from the World Development Indicators. This variable captures the priority assigned to health in public budgets and reflects the government’s commitment to financing healthcare services, public health programs, medical personnel, and service delivery capacity. Third, R&D expenditure is measured as research and development expenditure as a percentage of GDP, sourced from the World Development Indicators. It captures national research intensity and the broader scientific and technological environment that supports innovation, including medical technologies, diagnostic tools, and evidence-based health solutions. Overall, the three control variables are expected to positively influence health performance: GDP per capita through improved socioeconomic and fiscal capacity, government health expenditure through stronger health-service financing and coverage, and R&D expenditure through stronger innovation and technological capabilities that support better health outcomes.

Table 1 summarizes the descriptive statistics for the study variables across the panel of 19 developing countries from 2015 to 2023. It shows that the two health-performance indicators show moderate cross-country variation. Life expectancy at birth has an average value of 75.51 years, with a standard deviation of 4.56, ranging from 62.01 to 83.53 years. This indicates that the sampled countries differ considerably in long-term population health outcomes. The UHC service coverage index has a mean of 77.22, with values ranging from 59.00 to 88.00, suggesting that most countries have relatively broad essential health-service coverage, although important differences remain across countries and years. The AI Index has a mean of 0.0689 and a standard deviation of 0.1709, with values ranging from 0.0000 to 0.9943. This wide dispersion suggests substantial heterogeneity in national AI capabilities, with only a limited number of observations showing very high levels of AI development. The health innovation variable, measured by life science patents granted, shows the largest variation, with a mean of 4,856.49, a standard deviation of 12,187.46, and a range from 2.00 to 61,617.00. This reflects strong differences in life-science patenting activity across the sample. The DIGIT Index has an average of 0.5351, a standard deviation of 0.1883, and a range of 0.0000 to 0.9874. This indicates that digital infrastructure differs meaningfully across the sampled countries, which supports its use as a moderating variable. Regarding the controls, GDP per capita averages 11,962.08 constant 2015 US dollars, government health expenditure averages 11.6309% of general government expenditure, and R&D expenditure averages 1.0922% of GDP. Overall, the descriptive results show meaningful variation in health performance, AI capability, health innovation, digital infrastructure, and macroeconomic conditions, providing a suitable basis for testing the proposed mediation and moderation relationships.

**Table 1:** Variable measures and descriptive statistics.

Variable	Measure	Observations	Symbol	Mean	SD	Min	Max
Life expectancy at birth	Life expectancy at birth, total years	171	LE	75.51	4.56	62.01	83.53
UHC service coverage index	Coverage of essential health services	171	UHC	77.22	6.86	59.00	88.00
AI	AI Index	171	AI	0.0689	0.1709	0.0000	0.9943
Health innovation	Life science patents granted by the filing office	171	HIV	4,856.49	12,187.46	2.00	61,617.00
Digital infrastructure	Digital Infrastructure Index	171	DIGIT	0.5351	0.1883	0.0000	0.9874
GDP per capita	GDP per capita in constant 2015 US dollars	171	GDP	11,962.08	7,999.28	1,584.00	36,347.70
Government health expenditure	Domestic general government health expenditure (% of general government expenditure)	171	GHE	11.6309	3.8331	3.3762	19.3722
R&D expenditure	Research and development expenditure (% of GDP)	171	R&D	1.0922	1.0174	0.1170	4.9435

SD measures dispersion around the mean; Min indicates the lowest observed value; and Max indicates the highest observed value in the sample. Health innovation is reported in its original scale for descriptive purposes. However, due to the high dispersion and skewness of patent counts, the empirical estimates use the logarithmic transformation of this variable, ln(1 + life-science patents).

### 3.2 Methodology

This study employs Hayes’ conditional process framework to examine the direct, indirect, moderating, and moderated mediation effects linking AI, health innovation, digital infrastructure, and health performance. The empirical design is consistent with the conceptual model of the study, in which AI is treated as the principal explanatory variable, health innovation is introduced as the mediating mechanism, and digital infrastructure is incorporated as a conditioning factor that shapes both the AI–health innovation pathway and the direct AI–health performance relationship. Since health performance is measured using two alternative indicators, namely life expectancy at birth and the UHC service coverage index, the same empirical framework is estimated separately for each dependent variable. The empirical analysis proceeds sequentially. First, the study estimates the direct effect of AI on health performance. Second, it applies the Hayes PROCESS Model 4 to confirm the mediating role of health innovation. Third, it extends the analysis to Hayes PROCESS Model 8, which is appropriate when the moderator affects both the path from the independent variable to the mediator and the direct path from the independent variable to the outcome. This sequence allows the study to first establish whether health innovation transmits the AI–health performance relationship before assessing whether this mediation mechanism and the direct AI effect are conditional on digital infrastructure.

The first stage estimates the direct effect of AI on health performance before introducing the mediating and moderating variables. This baseline specification allows the study to assess whether national AI capability is directly associated with better health performance. The direct-effect model is expressed in Equation 1 as follows:


$$HP_{\mathrm{it}}=\alpha_{0}+\mathrm{cA}I_{\mathrm{it}}+\sum \alpha_{k}\text{Control}s_{\mathrm{it}}+\varepsilon_{\mathrm{it}}\;$$ (1)

Where $HP_{\mathrm{it}}$ denotes health performance, measured alternatively by life expectancy at birth and the UHC service coverage index. $AI_{\mathrm{it}}$ represents the AI Index, while $\text{Control}s_{\mathrm{it}}$ refers to the vector of control variables, including GDP per capita, domestic general government health expenditure, and R&D expenditure. The coefficient c captures the total effect of AI on health performance before accounting for mediation. A positive and statistically significant value of c would indicate that higher AI capability is associated with improved health performance.

The second stage examines the mediating effect of health innovation in the relationship between AI and health performance. This step first estimates whether AI contributes to health innovation and then assesses whether health innovation improves health performance after controlling for AI. The mediation (Equations 2, 3) are specified as follows:


$$\;\;\mathrm{HI}V_{\mathrm{it}}=\beta_{0}+\mathrm{aA}I_{\mathrm{it}}+\sum \beta_{k}\text{Control}s_{\mathrm{it}}+e_{\mathrm{Mit}}\;$$ (2)

$$\;\;HP_{\mathrm{it}}=\gamma_{0}+c^{\prime }AI_{\mathrm{it}}+\mathrm{bHI}V_{\mathrm{it}}+\sum \gamma_{k}\text{Control}s_{\mathrm{it}}+e_{\mathrm{Yit}}\;$$ (3)

Where $\;\;\mathrm{HI}V_{\mathrm{it}}$ denotes health innovation, measured by life science patents granted by filing office. The coefficient a measures the effect of AI on health innovation, while b captures the effect of health innovation on health performance after controlling for AI. The coefficient c′ represents the remaining direct effect of AI on health performance once health innovation is included in the model. The indirect effect of AI on health performance through health innovation in Equation 4 as:


IE=a×b (4)

A statistically significant indirect effect indicates that health innovation transmits part of the effect of AI to health performance. In this case, AI improves health performance not only directly, but also indirectly by stimulating health-related technological innovation.

The third stage introduces digital infrastructure as a moderating variable. In Hayes PROCESS Model 8, digital infrastructure moderates both the path from AI to health innovation and the direct path from AI to health performance. This makes it possible to test whether the innovation-enhancing and health-improving effects of AI are stronger in countries with more developed digital infrastructure. The moderated (Equations 5, 6) are specified as follows:


$$\begin{array}{l} \mathrm{HI}V_{\mathrm{it}}=\beta_{0}+\alpha_{1}AI_{\mathrm{it}}+\alpha_{2}\text{DIGI}T_{\mathrm{it}}+\alpha_{3}(AI_{\mathrm{it}}\times \text{DIGI}T_{\mathrm{it}})\\ +\sum \beta_{k}\text{Control}s_{\mathrm{it}}+e_{\mathrm{Mit}}\; \end{array}$$ (5)

$$\begin{array}{l} \;HP_{\mathrm{it}}=\gamma_{0}+c^{\prime }AI_{\mathrm{it}}+\mathrm{bHI}V_{\mathrm{it}}+\text{dDIGI}T_{\mathrm{it}}\\ +q(AI_{\mathrm{it}}\times \text{DIGI}T_{\mathrm{it}})+\sum \gamma_{k}\text{Control}s_{\mathrm{it}}+e_{\mathrm{Yit}}\; \end{array}$$ (6)

Where $\text{DIGI}T_{\mathrm{it}}$ ​ denotes the Digital Infrastructure Index. In the first equation, $\alpha_{1}$ ​ captures the effect of AI on health innovation when digital infrastructure is held constant, while $\alpha_{2}$ ​ measures the direct association between digital infrastructure and health innovation. The interaction term $AI_{\mathrm{it}}\times \text{DIGI}T_{\mathrm{it}}$ tests whether digital infrastructure changes the strength of the relationship between AI and health innovation. The coefficient $\alpha_{3}$ ​ is therefore the key parameter for testing the first moderation effect. A positive and statistically significant $\alpha_{3}$ ​ indicates that AI stimulates health innovation more strongly when digital infrastructure is higher. In the second equation, b measures the effect of health innovation on health performance, while q captures the moderating impact of digital infrastructure on the direct relationship between AI and health performance. A positive and statistically significant q indicates that the direct effect of AI on health performance becomes stronger in countries with more advanced digital infrastructure.

The fourth stage estimates the moderated mediation effect. Since digital infrastructure moderates the first-stage path from AI to health innovation, the indirect impact of AI on health performance through health innovation is no longer constant. Instead, it varies according to the level of digital infrastructure. The conditional indirect effect is expressed in Equation 7 as follows:


$$\text{Conditional indirect effect}=(\alpha_{1}+\alpha_{3}\text{DIGI}T_{\mathrm{it}})b$$ (7)

This expression shows that the mediating role of health innovation depends on the level of digital infrastructure. When digital infrastructure is high, the effect of AI on health innovation is expected to become stronger, thereby increasing the indirect effect of AI on health performance. Conversely, when digital infrastructure is weak, the ability of AI to generate health innovation may be constrained, reducing the strength of the indirect pathway. The index of moderated mediation is calculated in Equation 8 as follows:


$$\text{Index of moderated mediation}=\alpha_{3}b$$ (8)

This index indicates whether the indirect impact of AI on health performance through health innovation changes significantly across different levels of digital infrastructure. If the bootstrap confidence interval of this index does not include zero, the moderated mediation hypothesis is supported. This would mean that digital infrastructure significantly conditions the mediating role of health innovation in the AI–health performance relationship.

In addition to the conditional indirect effect, Model 8 also allows the direct effect of AI on health performance to vary according to digital infrastructure. The conditional direct effect is expressed in Equation 9 as follows:


$$\text{Conditional direct effect}=c^{\prime }+\text{qDIGI}T_{\mathrm{it}}$$ (9)

This expression indicates that the direct effect of AI on health performance depends on the level of digital infrastructure. If q is positive and significant, AI contributes more strongly to health performance in countries with stronger digital foundations. This is theoretically important because AI may improve health performance not only through innovation, but also through direct improvements in healthcare delivery, public health surveillance, telemedicine, diagnostic support, patient monitoring, administrative efficiency, and resource allocation.

The total conditional effect of AI on health performance combines the conditional direct effect and the conditional indirect effect through health innovation. It is expressed in Equation 10 as follows:


$$\begin{array}{l} \text{Total conditional effect}=(c^{\prime }+\text{qDIGI}T_{\mathrm{it}})\\ +(\alpha_{1}+\alpha_{3}\text{DIGI}T_{\mathrm{it}})b \end{array}$$ (10)

This specification allows the study to evaluate the overall effect of AI on health performance at different levels of digital infrastructure. It therefore captures the full conditional process through which AI affects health outcomes, both directly and indirectly through health innovation. The statistical significance of the indirect and moderated indirect effects is assessed using bootstrapped confidence intervals. Bootstrapping is preferred because indirect effects are often not normally distributed, making conventional normal-theory tests less reliable. Accordingly, mediation and moderated mediation effects are considered statistically significant when their bootstrap confidence intervals do not include zero. Conditional effects are evaluated at low, medium, and high levels of digital infrastructure to determine whether the AI–health innovation–health performance pathway becomes stronger as digital infrastructure improves.

**Figure 2:**
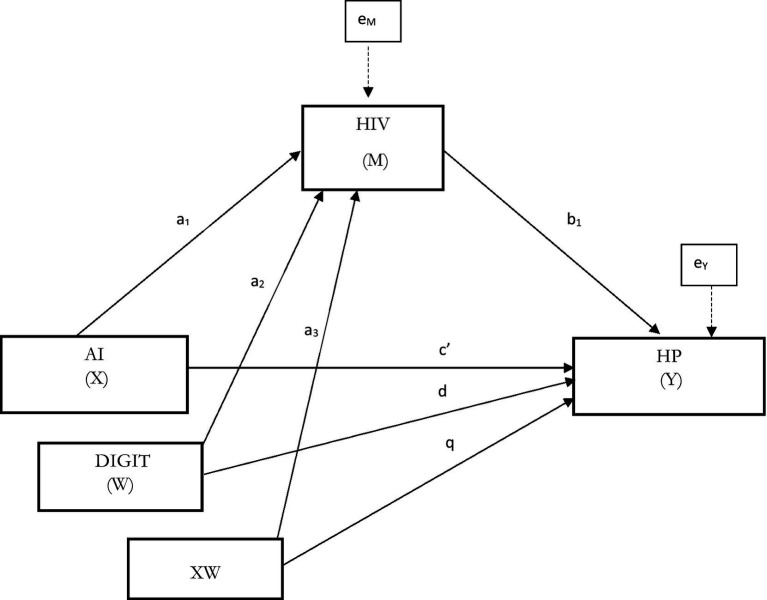
Statistical diagram: first-stage moderated mediation model.

Overall, Hayes’ PROCESS Model 8 provides a suitable empirical strategy for this study because it enables analysis of the full conditional process linking AI, health innovation, digital infrastructure, and health performance. As illustrated in Figure 2, the model specifies AI as the independent variable, health innovation as the mediating mechanism, and health performance as the outcome variable. The diagram also shows that digital infrastructure plays a dual moderating role by conditioning both the AI–health innovation pathway and the direct AI–health performance pathway. Accordingly, this framework allows the study to examine whether AI improves health performance directly, whether health innovation transmits this effect, whether digital infrastructure strengthens AI’s innovation-generating effect, and whether the indirect effect of AI through health innovation becomes stronger at higher levels of digital infrastructure.

For robustness, this study further applies the two-step SYS-GMM estimator to complement and validate the PROCESS-based mediation and moderated mediation results using an alternative dynamic panel identification strategy. This robustness check is introduced to address potential econometric concerns associated with panel data, including unobserved country-specific heterogeneity, dynamic persistence, simultaneity, reverse causality, heteroscedasticity, autocorrelation, and endogeneity. Health performance indicators, such as life expectancy and UHC service coverage, are likely to persist over time, while AI, health innovation, and digital infrastructure may be jointly determined with health performance. For example, AI may improve health outcomes, but countries with stronger health systems may also be better positioned to adopt AI technologies and generate health innovations. SYS-GMM is therefore appropriate because it combines first-difference equations with level equations and uses lagged values of potentially endogenous variables as internal instruments. Compared with difference GMM, SYS-GMM improves efficiency when the variables are persistent and when lagged levels are weak instruments for differenced variables. The two-step version is preferred because it yields more efficient estimates, while robust finite-sample-corrected standard errors are used to reduce downward bias in the estimated standard errors. To reduce the risk of instrument proliferation, the instrument matrix is collapsed, and the lag depth is restricted. Year dummies are included to control for common shocks affecting all countries over time.

The dynamic specification used to test the total effect of AI on health performance is expressed as follows:


$$\begin{array}{l} HP_{\mathrm{it}}=\alpha_{0}+\mathrm{\rho H}P_{\mathrm{it}-1}+\mathrm{cA}I_{\mathrm{it}}+\sum \alpha_{k}\text{Control}s_{\mathrm{it}}\\ +\mu_{i}+\lambda_{t}+\varepsilon_{\mathrm{it}} \end{array}$$ (11)

Where HP denotes health performance, measured alternatively by life expectancy at birth and UHC service coverage. HP_it − 1_ is the lagged dependent variable, AI represents AI, and Controls is the vector of control variables, including GDP per capita, government health expenditure, and R&D expenditure. μ_i_ captures unobserved country-specific effects, λ_t_ controls for common time shocks, and ε_it_ is the idiosyncratic error term. In this equation, c measures the total effect of AI on health performance.

To examine the mediating role of health innovation, the empirical analysis follows a two-equation mediation structure. The first equation estimates the effect of AI on health innovation:


$$\begin{array}{l} \mathrm{HI}V_{\mathrm{it}}=\delta_{0}+\emptyset \mathrm{HI}V_{\mathrm{it}-1}+\mathrm{aA}I_{\mathrm{it}}+\sum \alpha_{k}\text{Control}s_{\mathrm{it}}\\ +\mu_{i}+\lambda_{t}+\mu_{\mathrm{it}} \end{array}$$ (12)

Where HIV denotes health innovation, measured by life-science patents granted by the filing office. HIV_it − 1_ captures the dynamic persistence of health innovation. In this equation, a measures the effect of AI on health innovation. A positive and statistically significant a indicates that AI stimulates health innovation, satisfying the first condition for the proposed mediating mechanism.

The second mediation equation estimates the effect of health innovation on health performance after controlling for AI and digital infrastructure:


$$\begin{array}{l} HP_{\mathrm{it}}=\alpha_{1}+\mathrm{\rho H}P_{\mathrm{it}-1}+c\prime AI_{\mathrm{it}}+\mathrm{bHI}V_{\mathrm{it}}\\ +\sum \alpha_{k}\text{Control}s_{\mathrm{it}}+\mu_{i}+\lambda_{t}+\varepsilon_{\mathrm{it}} \end{array}$$ (13)

Where b measures the effect of health innovation on health performance, while c′ represents the direct effect of AI after accounting for the mediator. Mediation is supported if AI significantly affects health performance in Equation 11, AI significantly affects health innovation in Equation 12, and health innovation significantly affects health performance in Equation 13. If c′ remains statistically significant but becomes smaller than c, the results indicate partial mediation. If c′ becomes statistically insignificant after including health innovation, the results indicate full mediation.

To test the moderating role of digital infrastructure in the AI–health innovation pathway, the mediator equation is extended by adding digital infrastructure and its interaction with AI:


$$\begin{array}{l} \mathrm{HI}V_{\mathrm{it}}=\delta_{0}+\emptyset \mathrm{HI}V_{\mathrm{it}-1}+\alpha_{1}AI_{\mathrm{it}}+\alpha_{2}\text{DIGI}T_{\mathrm{it}}\\ +\alpha_{3}(AI_{\mathrm{it}}\times \text{DIGI}T_{\mathrm{it}})+\sum \alpha_{k}\text{Control}s_{\mathrm{it}}+\mu_{i}+\lambda_{t}+\mu_{\mathrm{it}} \end{array}$$ (14)

Where DIGITit denotes digital infrastructure. The interaction term AIit × DIGITit tests whether digital infrastructure strengthens the effect of AI on health innovation. The coefficient a3 is the key parameter for the moderation effect. A positive and statistically significant a3 indicates that digital infrastructure enhances the innovation effect of AI. To reduce multicollinearity and improve interpretation, the interaction term is constructed after mean-centering the AI and digital infrastructure variables.

The moderated mediation effect is evaluated by combining the conditional AI–health innovation effect from Equation 14 with the health innovation–health performance effect from Equation 13. This means that the indirect impact of AI on health performance through health innovation varies according to the level of digital infrastructure. The moderated mediation mechanism is supported when the interaction term AI_it_ × DIGIT_it_ significantly predicts health innovation in Equation 14, health innovation significantly predicts health performance in Equation 13, and the conditional indirect effect becomes stronger at higher levels of digital infrastructure.

In estimating the above models, the lagged dependent variable is treated as endogenous. AI, health innovation, digital infrastructure, and the interaction term are also treated as potentially endogenous or predetermined because they may be jointly determined with health performance. Their lagged values are therefore used as internal instruments. The control variables are included to isolate the effects of AI, health innovation, and digital infrastructure from broader macroeconomic and health-system conditions. The reliability of the SYS-GMM results is verified through several post-estimation diagnostic tests. The overall relevance of the explanatory variables is first examined using the Wald test, which assesses their joint statistical significance. Instrument validity is then evaluated through the Hansen test of over-identifying restrictions; an insignificant Hansen statistic suggests that the instruments are appropriate and uncorrelated with the error term. In addition, the Arellano–Bond (61) tests for serial correlation are applied to the differenced residuals. While the AR(1) statistic is normally expected to be significant due to the nature of first differencing, the AR(2) statistic should remain insignificant, confirming the absence of second-order serial correlation and supporting the validity of the moment conditions. Together, these diagnostic checks ensure that the two-step SYS-GMM estimates provide reliable evidence on the mediation and moderated mediation mechanisms proposed in the study.

The use of Hayes’ PROCESS macro as the primary empirical framework is justified by the study’s conditional process nature. The objective is not only to estimate the direct association between AI and health performance, but also to examine whether health innovation mediates this relationship and whether digital infrastructure conditions both the AI–health innovation pathway and the direct AI–health performance pathway. Hayes’ PROCESS macro is specifically designed to estimate mediation, moderation, and moderated mediation models within an integrated framework and to assess indirect and conditional indirect effects using bootstrapped confidence intervals (13, 54). This feature is particularly useful for the present study because PROCESS Model 4 allows estimation of the mediation pathway, while PROCESS Model 8 allows estimation of the first-stage moderated mediation structure, in which digital infrastructure moderates the effect of AI on health innovation and the direct effect of AI on health performance. Moreover, the use of PROCESS or conditional process analysis in panel-data contexts is not uncommon in recent empirical research in economics, sustainability, innovation, and development studies. For example, Nguyen and Tran (55) used PROCESS Model 4 as part of their analysis of panel data from developing economies, while Hao et al. (56), and Xie and Bruce (57) employed mediation, moderated mediation, or conditional process frameworks in panel-data settings. These studies support the use of PROCESS as a theory-driven tool for estimating mechanisms and boundary conditions. Therefore, we recognize that PROCESS does not, by itself, fully account for repeated country-year observations, unobserved country-specific heterogeneity, common time shocks, dynamic persistence, or within-country dependence. For this reason, the PROCESS-based results are complemented by two-step SYS-GMM estimation. This combined strategy allows PROCESS to test the hypothesized conditional mechanism directly and transparently, while SYS-GMM verifies whether the mediation and moderated mediation logic remains robust after accounting for panel-data concerns such as unobserved heterogeneity, dynamic persistence, simultaneity, reverse causality, and potential endogeneity.

## 4 Results and discussion

### 4.1 Conditional process analysis

The analysis first examines whether AI directly improves health performance. It then tests whether health innovation mediates this relationship using Hayes’ PROCESS Model 4. Finally, Hayes’ PROCESS Model 8 is applied to assess whether digital infrastructure strengthens both the AI–health innovation pathway and the direct AI–health performance relationship. Since health performance is measured by life expectancy and UHC service coverage, the same analytical sequence is estimated separately for each outcome. This approach allows the study to identify whether AI improves health performance, whether this effect operates through health innovation, and whether the strength of these relationships depends on the level of digital infrastructure.

To assess both the direct and indirect effects of AI on health performance, the study employs a simple mediation approach using Model 4 of Hayes’s PROCESS macro. This specification estimates the direct influence of AI on health performance while simultaneously capturing the indirect pathway through health innovation. Since health performance is measured using two alternative indicators, life expectancy and UHC service coverage, the mediation model is estimated separately for each dependent variable. Regarding life expectancy, the results in Table 2 show that AI exerts a positive and statistically significant total effect. The estimated total effect is 0.111, with a standard error of 0.022, a *p*-value of 0.000, and a 95% confidence interval of [0.068; 0.155]. Since this confidence interval does not include zero, the result supports H1, indicating that AI improves life expectancy in the sampled developing countries. More specifically, the direct effect of AI on life expectancy after accounting for health innovation is also positive and statistically significant, with a coefficient of 0.046, a standard error of 0.023, a *p*-value of 0.046, and a 95% confidence interval of [0.001; 0.091]. This means that AI continues to improve life expectancy even after accounting for the mediating role of health innovation. Substantively, this result indicates that stronger national AI capability is associated with better long-term population health outcomes. This direct relationship can be explained through several clinical, managerial, and public-health mechanisms. AI can improve diagnostic accuracy, support clinical decision-making, strengthen patient monitoring, enhance disease prediction, improve hospital management, and support more efficient resource allocation. AI can also improve public health surveillance and early-warning systems, allowing health authorities to detect health risks earlier and respond more effectively. These functions are particularly important in developing countries, where health systems often face shortages of skilled professionals, fragmented information systems, and limited service-delivery capacity. Therefore, AI appears to improve life expectancy by strengthening the ability of health systems to prevent, detect, and manage disease more efficiently. This finding is consistent with Al Kuwaiti et al. (15) and Olawade et al. (16), who show that AI improves healthcare and public health through diagnosis, monitoring, surveillance, and health-risk prediction. It also supports Fisher and Rosella (19), who argue that AI can improve public health decision-making when supported by organizational readiness, ethical governance, and data capacity.

**Table 2:** Direct and indirect effects of AI on life expectancy.

Variables	Health innovation (M)					Life expectancy (Y)				
	Path	Coef.	SE	*p*	95%CI	Path	Coef.	SE	*p*	95%CI
AI (X)	a_1_	0.529	0.076	0.000	[0.379; 0.678]	c’	0.046	0.023	0.046	[0.001; 0.091]
Health innovation(M)						b_1_	0.124	0.021	0.000	[0.083; 0.164]
GDP per capita	a_4_	0.090	0.041	0.013	[0.040; 0.140]	b_4_	0.066	0.021	0.092	[0.026; 0.106]
Government health expenditure	a_5_	0.077	0.034	0.001	[0.056; 0.098]	b_5_	0.123	0.039	0.000	[0.055; 0.191]
R&D expenditure	a_6_	0.106	0.015	0.000	[0.076; 0.135]	b_6_	−0.009	0.005	0.050	[−0.018; −0.000]
Constant	$\beta_{0}$	1.295	0.165	0.000	[0.968; 1.621]	$\gamma_{0}$	3.599	0.051	0.000	[3.498; 3.700]
R	0.737					0.832				
R^2^	0.521					0.692				
F	49.277 (0.000)					74.316 (0.000)				
Total effect of AI on life expectancy										
		Coef.		SE	*p*-value		95% CI			
Total effect		0.111		0.022	0.000		[0.068; 0.155]			
Direct effect		0.046		0.023	0.046		[0.001; 0.091]			
Indirect effect		0.065		0.016	—		[0.036; 0.099]			

X is AI; M is health innovation; Y is the dependent variable; Coef. indicates coefficient; SE is standard error; CI is confidence interval. The indirect effect is assessed using bootstrap confidence intervals; significance is supported when the interval excludes zero.

**Figure 3:**
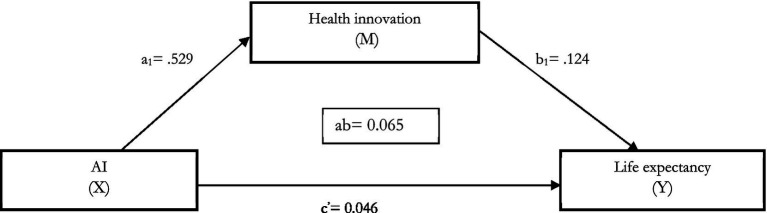
Simple mediating effect of health innovation on o AI–Life expectancy nexus.

The mediation analysis then examines whether AI contributes to life expectancy through health innovation. As shown in Table 2 and Figure 3, the first component of this indirect pathway is the impact of AI on health innovation, represented by a1. The coefficient is positive and statistically significant, with a1 = 0.529, a standard error of 0.076, a *p*-value of 0.000, and a 95% confidence interval of [0.379; 0.678]. Since this confidence interval excludes zero, the result confirms that AI significantly promotes health innovation. This means that countries with stronger AI capabilities are more likely to generate health-related and life-science innovations. This relationship can be explained by AI’s capacity to process large biomedical datasets, improve knowledge discovery, accelerate medical research, support diagnostic development, assist drug discovery, and improve the design of medical technologies. AI can also help researchers and health institutions identify disease patterns, test predictive models, and develop new health solutions more efficiently. Therefore, AI serves as an upstream digital capability that strengthens the knowledge base underpinning health innovation. This finding is consistent with Topol (8) and Rajpurkar et al. (7), who emphasize the role of AI in medical imaging, diagnosis, biomedical research, and clinical decision support. It also supports Arora (28) and da Silva (29), who view AI as a major source of digital healthcare innovation, especially because it improves the production and application of biomedical knowledge.

The second component of the mediation pathway concerns the effect of health innovation on life expectancy, represented by b1. As reported in Table 2, b1 is positive and statistically significant, with b1 = 0.124, a standard error of 0.021, a *p*-value of 0.000, and a 95% confidence interval of [0.083; 0.164]. Since this interval does not include zero, the result confirms that health innovation significantly improves life expectancy. This means that countries with stronger life-science patenting and medical technology development are better positioned to improve population survival. This relationship can be explained through several mechanisms. Health innovation improves diagnostic capacity, treatment quality, disease prevention, patient monitoring, and healthcare efficiency. Medical technologies and life-science innovations can support earlier detection of diseases, improve the management of chronic conditions, reduce preventable mortality, and expand the technological capacity of health systems. Therefore, life expectancy depends not only on health expenditure and service access, but also on the availability of innovative health technologies that improve the effectiveness and quality of care. This finding is consistent with Moreira et al. (36), who show that innovation improves healthcare organizational performance, and with Madden et al. (35), who confirm that healthcare innovation enhances efficiency, quality, safety, and clinical outcomes. It also supports Van Nuys et al. (39) and Muradov et al. (40), who show that medical innovation contributes to longer life expectancy. Furthermore, the indirect effect reported in Table 2 confirms the mediating role of health innovation in the AI–life expectancy relationship. The estimated indirect effect, represented by a1 × b1, is positive and statistically significant, with a value of 0.065 and a bootstrap confidence interval of [0.036; 0.099]. Since the confidence interval excludes zero, the indirect impact is statistically significant. This result supports H2 for life expectancy, indicating that AI improves life expectancy partly by stimulating health innovation. As illustrated in Figure 3, this indirect effect results from two significant relationships: AI increases health innovation, and health innovation, in turn, improves life expectancy. However, because the direct effect of AI remains significant, the mediation is partial rather than full. This implies that AI improves life expectancy through two complementary routes: a direct health-system route and an indirect innovation route.

**Figure 4:**
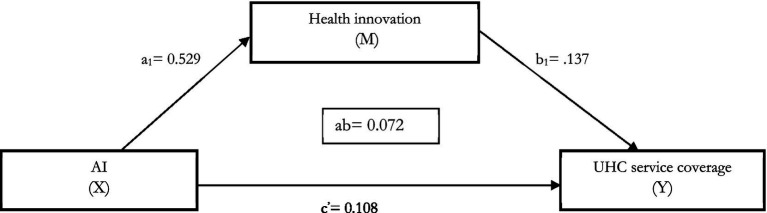
Simple mediating effect of health innovation on o AI–UHC service coverage nexus.

Table 3 reports similar evidence when health performance is measured by UHC service coverage. The total effect of AI on UHC is positive and statistically significant, with a coefficient of 0.181, a standard error of 0.031, a *p*-value of 0.000, and a 95% confidence interval of [0.120; 0.242]. The direct effect of AI on UHC after accounting for health innovation is also positive and significant, with a coefficient of 0.108, a standard error of 0.033, a *p*-value of 0.001, and a confidence interval of [0.043; 0.174]. This result indicates that AI directly improves UHC service coverage. The mechanism behind this direct effect is that AI can help health systems improve service planning, identify underserved populations, optimize resource allocation, support telemedicine, improve patient triage, and strengthen service-delivery coordination. These functions can expand the coverage and quality of essential health services, particularly in countries where health systems face resource constraints and unequal access. The mediation pathway is also confirmed. AI significantly improves health innovation, with a coefficient of 0.529, while health innovation significantly improves UHC, with b1 = 0.137, a standard error of 0.030, a *p*-value of 0.000, and a confidence interval of [0.078; 0.196]. This means that health innovation contributes to broader essential health-service coverage by improving diagnostic tools, treatment technologies, medical devices, and health-system efficiency. The indirect effect of AI on UHC through health innovation is 0.072, with a bootstrap confidence interval of [0.049; 0.110], confirming that the mediation effect is statistically significant. Thus, H2 is also supported for UHC service coverage. As illustrated in Figure 4, AI contributes to UHC through both a direct service-delivery mechanism and an indirect innovation mechanism. These findings are consistent with Akinwale and AboAlsamh (37), who show that technological innovation improves healthcare performance, and with Hussain et al. (38), who link sustainable healthcare innovation to broader improvements in health systems. Overall, the results in Tables 2, 3 provide strong empirical support for the central argument of this study: AI improves health performance directly by enhancing healthcare delivery and public-health management, and indirectly by stimulating health innovation that strengthens life expectancy and UHC service coverage. This writing follows the same mediation-discussion direction as the attached example, which interprets the total, direct, and indirect effects through values, mechanisms, and prior literature comparison.

**Table 3:** Direct and indirect effects of AI on UHC service coverage.

Variables	Health innovation (M)					UHC service coverage (Y)				
	Coef.		SE	*p*	95%CI	Coef.		SE	*p*	95%CI
AI (X)	a_1_	0.529	0.076	0.000	[0.379; 0.678]	c’	0.108	0.033	0.001	[0.043; 0.174]
Health innovation(M)						b_1_	0.137	0.030	0.000	[0.078; 0.196]
GDP per capita	a_4_	0.090	0.041	0.013	[0.040; 0.140]	b_4_	0.095	0.009	0.000	[0.077; 0.112]
Government health expenditure	a_5_	0.077	0.034	0.001	[0.056; 0.098]	b_5_	0.049	0.013	0.000	[0.023; 0.074]
R&D expenditure	a_6_	0.106	0.015	0.000	[0.076; 0.135]	b_6_	−0.004	0.007	0.538	[−0.017; 0.009]
Constant	$\beta_{0}$	1.295	0.165	0.000	[0.968; 1.621]	$\gamma_{0}$	3.300	0.075	0.000	[3.152; 3.447]
R	0.737					0.840				
R^2^	0.521					0.706				
F	49.277 (0.000)					79.105 (0.000)				
Total effect of AI on UHC service coverage										
		Coeff.		S. E.	*p*-value		CI 95%			
Total effect		0.181		0.031	0.000		[0.120; 0.242]			
Direct effect		0.108		0.033	0.001		[0.043; 0.174]			
Indirect effect		0.072		0.016	—		[0.049; 0.110]			

X is AI; M is health innovation; Y is the dependent variable; Coef. indicates coefficient; SE is standard error; CI is confidence interval. The indirect effect is assessed using bootstrap confidence intervals; significance is supported when the interval excludes zero.

Table 4 reports the conditional effect of AI on health innovation and examines whether digital infrastructure moderates this relationship. The results show that AI has a positive and statistically significant effect on health innovation, with a coefficient of 0.586, a standard error of 0.032, a *p*-value of 0.000, and a 95% confidence interval of [0.523; 0.649]. Since the confidence interval does not include zero, this result confirms that AI directly strengthens health innovation. This indicates that countries with stronger AI capability are more likely to generate health-related inventions, life-science patents, and medical technology innovations. This effect can be explained by AI’s ability to improve biomedical data processing, accelerate medical research, support diagnostic discovery, assist drug development, and strengthen experimentation in health technologies. AI can also help researchers, firms, and health institutions identify disease patterns, develop predictive tools, optimize clinical workflows, and transform large-scale health data into new medical knowledge. Therefore, AI functions as an upstream digital capability that improves the knowledge base and technological capacity required for health innovation. Table 4 also shows that digital infrastructure has a positive and statistically significant direct effect on health innovation, with a coefficient of 0.484, a standard error of 0.015, a *p*-value of 0.000, and a 95% confidence interval of [0.454; 0.515]. This result indicates that countries with stronger digital infrastructure tend to achieve higher levels of health innovation. This can be explained by the fact that health innovation increasingly depends on digital connectivity, broadband access, secure data platforms, cloud-based systems, electronic health records, digital research networks, and interoperable information systems. These infrastructures allow health institutions and researchers to collect, exchange, store, and analyze health-related data more efficiently. In this sense, digital infrastructure is not only a technical background condition; it is an enabling system that supports the production and diffusion of medical technologies. The finding is consistent with Swan et al. (42), who argue that AI creates healthcare value through interaction with broader digital health systems, and with Feroz and Kwak (41), who emphasize that AI and digital transformation converge through digital platforms, data systems, and technology-enabled processes.

**Table 4:** Conditional effect of AI on health innovation (HIV).

Variables	Health innovation (M)				
	Path	Coef.	SE	*p*	95%CI
AI (X)	a_1_	0.586	0.032	0.000	[0.523; 0.649]
DIGIT (W)	a_2_	0.484	0.015	0.000	[0.454; 0.515]
AI * DIGIT (XW)	a_3_	0.897	0.080	0.000	[0.738; 1.056]
GDP per capita	a_4_	0.115	0.006	0.026	[0.103; 0.126]
Government health expenditure	a_5_	0.216	0.007	0.000	[0.203; 0.229]
R&D expenditure	a_6_	0.081	0.003	0.034	[0.075; 0.087]
Constant	$\beta_{0}$	3.884	0.044	0.000	[3.757; 3.932]
R	0.996				
R^2^	0.992				
F (HC4)	3831.511 (0.000)				
Test of the highest order unconditional interaction					
XW	R2-chng	F (HC4)	df1	df2	*p*-value
	0.010	124.444	1	164	0.000

AI is AI; HIV is health innovation; DIGIT is digital infrastructure; GDP is GDP per capita; GHE is government health expenditure; RD is R&D expenditure. Coef. indicates coefficient; SE indicates standard error; CI indicates confidence interval. HC4 heteroscedasticity-consistent standard errors were used. The interaction term AI × DIGIT tests whether digital infrastructure moderates the impact of AI on health innovation. Significance is supported when the 95% confidence interval excludes zero.

More importantly, the interaction between AI and digital infrastructure is positive and statistically significant, with a coefficient of 0.897, a standard error of 0.080, a *p*-value of 0.000, and a 95% confidence interval of [0.738; 1.056]. This indicates that digital infrastructure significantly moderates the relationship between AI and health innovation. In other words, the positive effect of AI on health innovation becomes stronger when digital infrastructure is higher. The test of the highest-order unconditional interaction further confirms this moderating effect, with an R^2^ change of 0.010, an F-statistic of 124.444, and a *p*-value of 0.000. This means that including the interaction term significantly improves the prediction of health innovation. The mechanism behind this result is that AI-driven health innovation depends heavily on data availability, digital connectivity, computing capacity, secure servers, and interoperable health-information systems. When digital infrastructure is weak, AI capabilities may remain concentrated in isolated research centers or pilot projects, limiting their ability to drive broader health innovation. Conversely, stronger digital infrastructure enables AI to be embedded in medical research, telemedicine systems, health data platforms, and digital innovation networks, thereby increasing its contribution to medical technology development. Thus, the result supports H3, which posits that digital infrastructure strengthens AI’s effect on health innovation.

**Figure 5:**
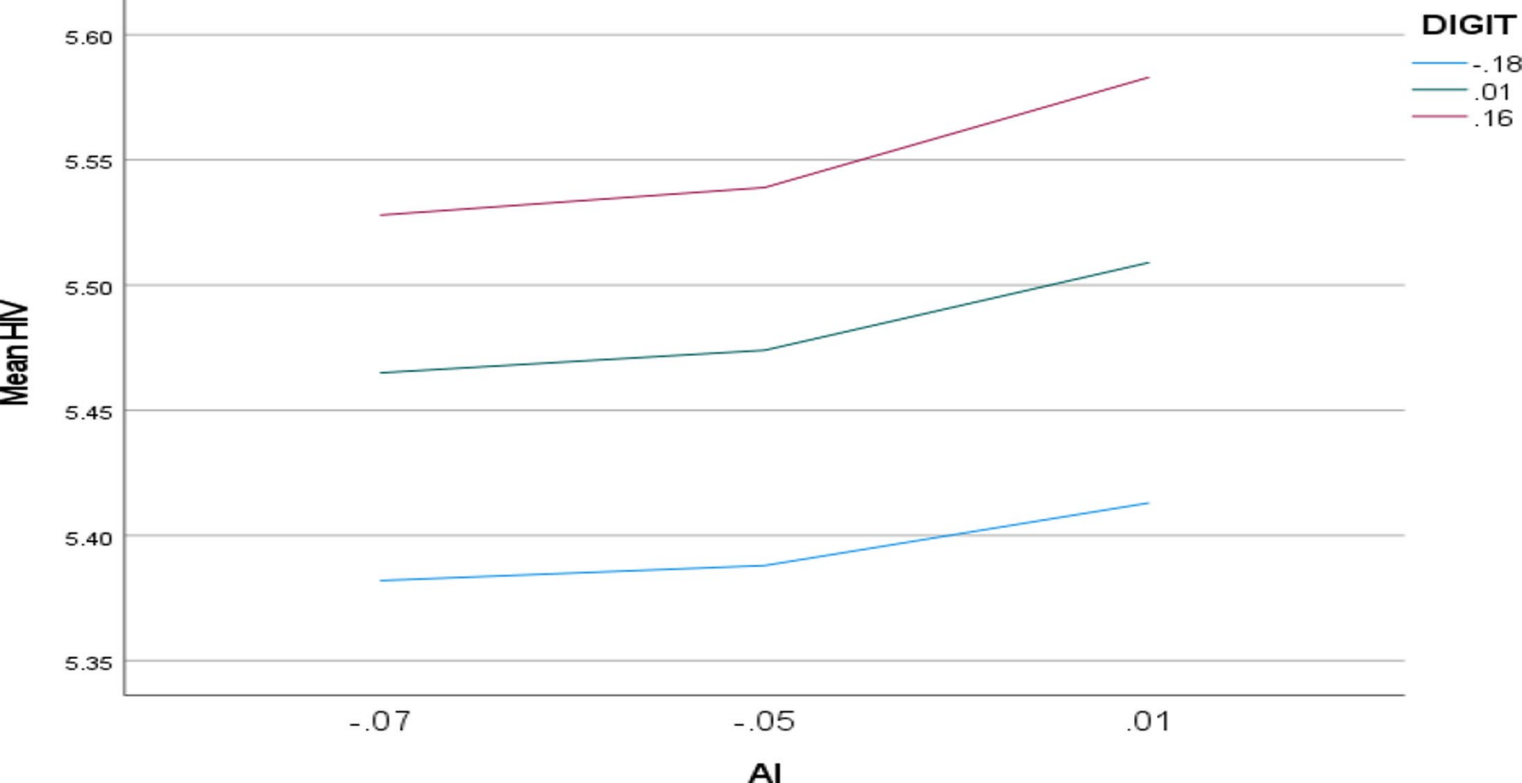
Conditional effect of AI on HIV at different levels of DIGIT.

The conditional effects reported in Table 5 and illustrated in Figure 5 provide a clearer interpretation of this moderating effect. When digital infrastructure is low, corresponding to the 16th percentile of DIGIT, the effect of AI on health innovation is positive and statistically significant, with an effect of 0.422, a standard error of 0.044, a *p*-value of 0.000, and a 95% confidence interval of [0.335; 0.510]. This means that AI still promotes health innovation under weak digital infrastructure conditions, but the magnitude of the effect is relatively limited. At the middle level of digital infrastructure, corresponding to the 50th percentile, the conditional effect increases to 0.597, with a standard error of 0.031, a *p*-value of 0.000, and a confidence interval of [0.535; 0.658]. When digital infrastructure is high, corresponding to the 84th percentile, the effect reaches 0.730, with a standard error of 0.023, a *p*-value of 0.000, and a confidence interval of [0.685; 0.776]. These results show a clear upward pattern: AI promotes health innovation at all levels of digital infrastructure, but its effect becomes progressively stronger as digital infrastructure improves. Figure 5 visually supports this result by showing that the slope linking AI to health innovation becomes steeper at higher levels of digital infrastructure. This means that the innovation-enhancing effect of AI is conditional on the digital environment in which AI is deployed. Substantively, this finding indicates that AI capabilities are more likely to be transformed into health innovation when countries have stronger digital foundations. AI-based innovation requires large volumes of reliable data, stable digital communication, interoperable platforms, and sufficient computing infrastructure. These conditions enable researchers and health institutions to apply AI to medical imaging, disease prediction, biomedical discovery, telemedicine, digital diagnostics, and clinical decision-support systems. Without these foundations, the innovation value of AI may be constrained because health systems lack the capacity to collect, process, and share data at scale. This result is consistent with Stoumpos et al. (14), who argue that digital transformation in healthcare requires infrastructure, workforce readiness, technology acceptance, and organizational change. It also supports Alami et al. (3) and World Health Organization (2), who emphasize that AI benefits in developing countries depend on data readiness, digital infrastructure, governance, and implementation capacity. Overall, Tables 4, 5 and Figure 5 confirm that digital infrastructure acts as an amplifying condition that enables AI to generate stronger health innovation outcomes.

**Table 5:** Conditional effects of AI on HIV at different levels of DIGIT.

Levels of DIGIT	DIGIT	Effect	SE (HC4)	*p*	LLCI	ULCI
Low (16th)	−0.183	0.422	0.044	0.000	0.335	0.510
Middle (50th)	0.012	0.597	0.031	0.000	0.535	0.658
High (84th)	0.161	0.730	0.023	0.000	0.685	0.776

AI is AI; HIV is health innovation; DIGIT is digital infrastructure. SE indicates HC4 heteroscedasticity-consistent standard error. LLCI and ULCI indicate the lower and upper limits of the 95% confidence interval, respectively. The conditional effects indicate the effect of AI on health innovation at different levels of digital infrastructure. Significance is supported when the 95% confidence interval excludes zero.

Table 6 reports the conditional effects of AI on health performance, measured by life expectancy and UHC service coverage. For life expectancy, the results show that AI has a positive and statistically significant direct effect, with a coefficient of 0.364, a standard error of 0.017, a *p*-value of 0.000, and a 95% confidence interval of [0.331; 0.397]. Since the confidence interval does not include zero, the result confirms that AI directly improves life expectancy after accounting for health innovation, digital infrastructure, the interaction term, and the control variables. This indicates that stronger national AI capability is associated with better long-term population health outcomes. The mechanism behind this direct effect is that AI can improve diagnostic accuracy, support clinical decision-making, enhance disease prediction, strengthen patient monitoring, optimize hospital management, and improve the allocation of scarce health resources. These functions are particularly relevant for developing countries, where health systems often face shortages of skilled professionals, uneven access to care, and weak information systems. Therefore, AI can contribute to life expectancy by strengthening the capacity of health systems to prevent, detect, and manage diseases more effectively. The results also show that health innovation has a positive and statistically significant effect on life expectancy, with a coefficient of 0.047, a standard error of 0.018, a *p*-value of 0.010, and a 95% confidence interval of [0.012; 0.082]. This result means that health innovation continues to improve life expectancy even after digital infrastructure and the AI–DIGIT interaction is included in the model. This can be explained by the role of medical technologies, diagnostic devices, biotechnology, and life-science innovations in improving disease prevention, treatment quality, patient monitoring, and clinical outcomes. Digital infrastructure also has a positive and statistically significant effect on life expectancy, with a coefficient of 0.251, a standard error of 0.009, a *p*-value of 0.000, and a confidence interval of [0.243; 0.269]. This indicates that stronger digital infrastructure is directly associated with better life-expectancy outcomes. Digital infrastructure can improve population health by enabling electronic health records, telemedicine, remote consultation, digital surveillance, health-data exchange, and more coordinated service delivery. These mechanisms allow health systems to identify health risks earlier, respond more quickly, and improve continuity of care. Most importantly, the interaction between AI and digital infrastructure is positive and statistically significant for life expectancy, with a coefficient of 0.814, a standard error of 0.036, and a 95% confidence interval of [0.743; 0.886]. This confirms that digital infrastructure significantly moderates the relationship between AI and life expectancy. In other words, AI contributes more strongly to life expectancy when digital infrastructure is higher. The test of the highest-order unconditional interaction confirms this conclusion, with an R^2^ change of 0.014, an F-statistic of 512.255, and a *p*-value of 0.000. This means that the interaction between AI and digital infrastructure significantly improves the prediction of life expectancy. This result supports H4 for life expectancy. Mechanistically, AI-based diagnosis, patient monitoring, disease surveillance, and resource allocation require reliable digital foundations to operate effectively. When digital infrastructure is weak, AI tools may be used only in limited institutions, reducing their system-wide impact. When digital infrastructure is strong, AI can be integrated into national health-information systems, telemedicine platforms, hospitals, and public-health networks, allowing its benefits to reach more patients and institutions.

**Table 6:** Conditional effects of AI on health performance (LE & UHC).

Variables	Life expectancy (LE)					UHC service coverage (UHC)				
	Path	Coef.	SE	*p*	95%CI	Path	Coef.	SE	*p*	95%CI
AI (X)	c’	0.364	0.017	0.000	[0.331; 0.397]	c’	0.391	0.011	0.000	[0.369; 0.414]
Health innovation (HIV)	b_1_	0.047	0.018	0.010	[0.012; 0.082]	b_1_	0.068	0.014	0.011	[0.040; 0.097]
DIGIT (W)	d	0.251	0.009	0.000	[0.243; 0.269]	d	0.291	0.007	0.000	[0.277; 0.306]
AI * DIGIT (XW)	q	0.814	0.036		[0.743; 0.886]	q	0.890	0.030	0.000	[0.831; 0.949]
GDP per capita	a_4_	0.031	0.002	0.003	[0.026; 0.035]	a_4_	0.026	0.002	0.022	[0.022; 0.029]
Government health expenditure	a_5_	0.057	0.004	0.000	[0.049; 0.065]	a_5_	0.051	0.003	0.042	[0.044; 0.058]
R&D expenditure	a_6_	0.021	0.002	0.021	[0.018; 0.024]	a_6_	0.020	0.001	0.019	[0.017; 0.022]
Constant	$\beta_{0}$	3.632	0.068	0.000	[3.497; 3.766]	$\gamma_{0}$	3.621	0.056	0.000	[3.510; 3.732]
R	0.999					0.948				
R^2^	0.991					0.903				
F (HC4)	12573.639 (0.000)					17741.475 (0.000)				
Test of the highest order unconditional interaction										
XW	R2-chng	F (HC4)	df1	df2	*p*-value	R2-chng	F (HC4)	df1	df2	*p*-value
	0.014	512.255	1	163	0.000	0.014	893.005	1	163	0.000

LE is life expectancy; UHC is UHC service coverage; AI is AI; HIV is health innovation; DIGIT is digital infrastructure; GDP is GDP per capita; GHE is government health expenditure; RD is R&D expenditure. Coef. indicates coefficient; SE indicates standard error; CI indicates confidence interval. HC4 heteroscedasticity-consistent standard errors were used. The interaction term AI × DIGIT tests whether digital infrastructure moderates the direct effect of AI on health performance. Significance is supported when the 95% confidence interval excludes zero.

The same pattern is observed when health performance is measured by UHC service coverage. Table 6 shows that AI has a positive and statistically significant direct effect on UHC, with a coefficient of 0.391, a standard error of 0.011, a *p*-value of 0.000, and a 95% confidence interval of [0.369; 0.414]. This result indicates that AI directly improves the coverage of essential health services. The mechanism behind this relationship is that AI can improve service planning, identify underserved populations, optimize resource allocation, support digital triage, expand telemedicine, improve referral systems, and strengthen health-service monitoring. These functions are central to UHC because universal health coverage depends not only on the existence of health services but also on the ability of health systems to deliver them efficiently, equitably, and continuously. The results also show that health innovation positively affects UHC service coverage, with a coefficient of 0.068, a standard error of 0.014, a *p*-value of 0.011, and a confidence interval of [0.040; 0.097]. This means that health innovation contributes to broader health-service coverage by improving diagnostic tools, treatment technologies, medical devices, and digital health solutions. Digital infrastructure also has a positive and significant effect on UHC service coverage, with a coefficient of 0.291, a standard error of 0.007, a *p*-value of 0.000, and a 95% confidence interval of [0.277; 0.306]. This result indicates that countries with stronger digital infrastructure tend to have better essential health-service coverage. The mechanism is that digital infrastructure improves connectivity between patients, providers, and institutions; supports telemedicine and remote care; facilitates electronic health records; and allows health systems to monitor service gaps more effectively. More importantly, the interaction between AI and digital infrastructure is positive and statistically significant, with a coefficient of 0.890, a standard error of 0.030, a *p*-value of 0.000, and a confidence interval of [0.831; 0.949]. This confirms that digital infrastructure strengthens the effect of AI on UHC service coverage. The test of the highest-order interaction further supports this result, with an R^2^ change of 0.014, an F-statistic of 893.005, and a *p*-value of 0.000. Thus, H4 is also supported for UHC service coverage. This finding means that AI has a stronger effect on UHC when it operates within digitally advanced health systems.

**Figure 6:**
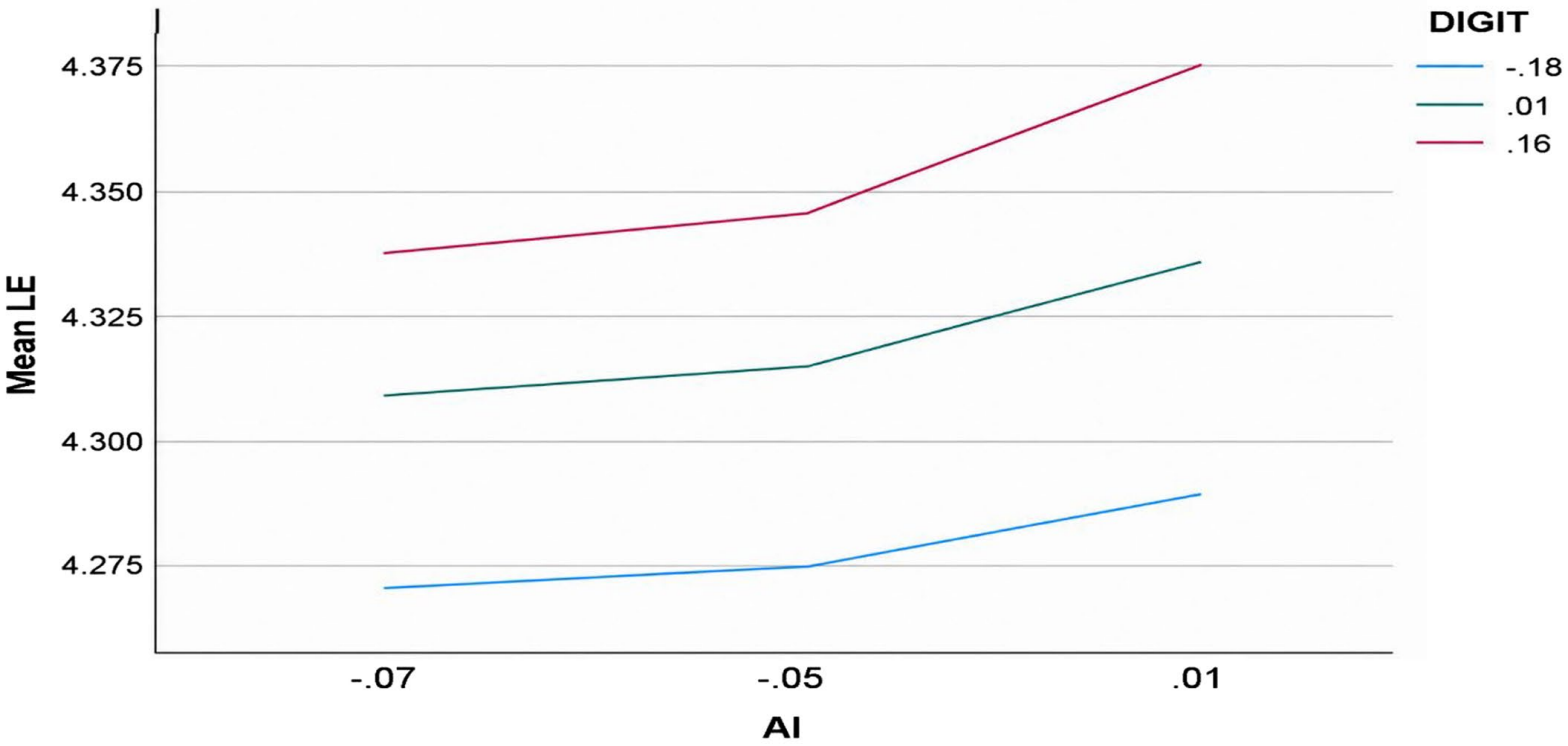
Conditional effect of AI on LE at different levels of DIGIT.

**Figure 7:**
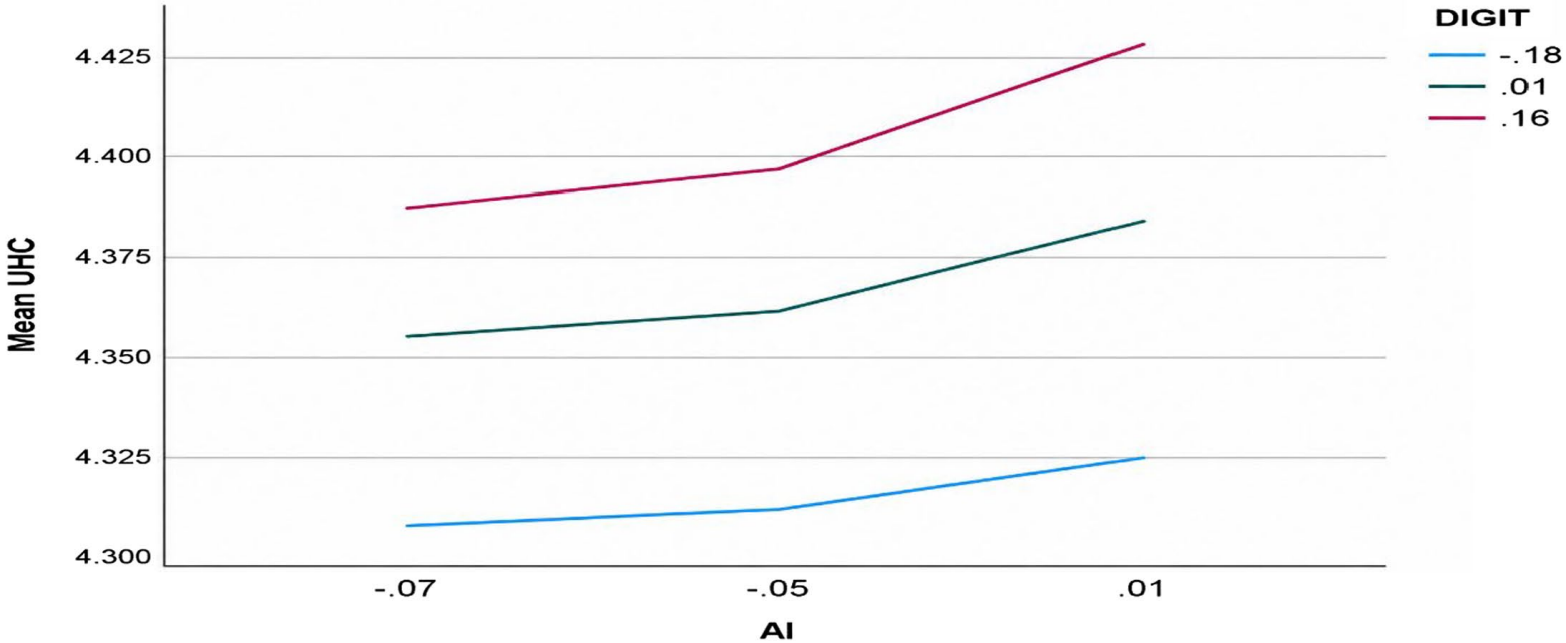
Conditional effect of AI on UHC at different levels of DIGIT.

The conditional effects reported in Table 7 and illustrated in Figures 6, 7 provide additional evidence of this moderating role. For life expectancy, when digital infrastructure is low, the effect of AI is positive and significant, with an effect of 0.215, a standard error of 0.020, a *p*-value of 0.000, and a 95% confidence interval of [0.176; 0.255]. This means that AI still improves life expectancy under weak digital infrastructure conditions, but the magnitude of the effect is relatively limited. At the middle level of digital infrastructure, the effect increases to 0.374, with a standard error of 0.017 and a confidence interval of [0.341; 0.407]. At the high level of digital infrastructure, the effect reaches 0.495, with a standard error of 0.016 and a confidence interval of [0.464; 0.526]. This upward pattern indicates that the contribution of AI to life expectancy becomes progressively stronger as digital infrastructure improves. Figure 6 visually confirms this result by showing that the slope linking AI to life expectancy becomes steeper at higher levels of digital infrastructure. For UHC service coverage, the conditional effects follow the same pattern. At the low level of digital infrastructure, the effect of AI on UHC is 0.229, with a standard error of 0.013, a *p*-value of 0.000, and a confidence interval of [0.202; 0.225]. At the middle level of digital infrastructure, the effect increases to 0.402, with a standard error of 0.011 and a confidence interval of [0.379; 0.424]. At the high level of digital infrastructure, the effect reaches 0.534, with a standard error of 0.012 and a confidence interval of [0.511; 0.558]. Figure 7 visually supports this result by showing a steeper AI–UHC relationship at higher levels of digital infrastructure. These results show that AI improves both life expectancy and UHC at all levels of digital infrastructure, but its effect is substantially stronger in countries with more advanced digital foundations. The increase from 0.215 to 0.495 for life expectancy and from 0.229 to 0.534 for UHC demonstrates that digital infrastructure acts as an amplifying condition for AI-based health improvements.

**Table 7:** Conditional effects of AI on health performance at different levels of DIGIT.

Levels of DIGIT	DIGIT	Life expectancy (LE)					UHC service coverage (UHC)				
		Effect	SE (HC4)	*p*	LLCI	ULCI	Effect	SE (HC4)	*p*	LLCI	ULCI
Low (16^th^)	−0.183	0.215	0.020	0.000	0.176	0.255	0.229	0.013	0.000	0.202	0.225
Middle (50^th^)	0.012	0.374	0.017	0.000	0.341	0.407	0.402	0.011	0.000	0.379	0.424
High (84^th^)	0.161	0.495	0.016	0.000	0.464	0.526	0.534	0.012	0.000	0.511	0.558

LE is life expectancy; UHC is UHC service coverage; AI is AI; DIGIT is digital infrastructure. SE indicates HC4 heteroscedasticity-consistent standard error. LLCI and ULCI indicate the lower and upper limits of the 95% confidence interval, respectively. The conditional effects show the effect of AI on health performance at different levels of digital infrastructure.

These findings support the argument that digital infrastructure is a critical enabling condition for transforming AI capability into health-performance gains. AI-based healthcare applications depend on data systems, broadband networks, digital platforms, secure servers, electronic medical records, and interoperable health-information systems. When these foundations are weak, AI may remain confined to isolated hospitals, research institutions, or pilot projects, limiting its impact on population-level health outcomes. By contrast, when digital infrastructure is robust, AI can be scaled across health systems and integrated into clinical care, public health surveillance, telemedicine, and service delivery platforms. This explains why AI produces stronger effects on life expectancy and UHC in digitally advanced contexts. These findings are consistent with the World Health Organization (2), which emphasizes that AI in health requires infrastructure, data governance, and system readiness. They also support Alami et al. (3), who argue that AI can improve health systems in low- and middle-income countries only when implementation capacity and digital readiness are sufficient. Similarly, Karami and Madlool (4) emphasize that the integration of AI and digital health is essential for strengthening health systems in developing countries. Overall, the results in Tables 4–7 and Figures 5–7 confirm that digital infrastructure plays a dual moderating role: it strengthens the effect of AI on health innovation and amplifies AI’s direct effect on health performance.

Tables 8, 9 examine the conditional indirect effects of AI on health performance through health innovation at different levels of digital infrastructure. This step is important because previous results indicate that AI improves health innovation and that health innovation improves health performance, while the moderated mediation analysis assesses whether this indirect pathway strengthens when digital infrastructure is higher. For life expectancy, Table 8 shows that the conditional indirect effect of AI through health innovation is positive and statistically significant at all three levels of digital infrastructure. When digital infrastructure is low, the indirect effect is 0.020, with a bootstrap standard error of 0.007 and a 95% bootstrap confidence interval of [0.005; 0.034]. Since this confidence interval does not include zero, the indirect effect is statistically significant. This means that even with weak digital infrastructure, AI can still indirectly improve life expectancy by stimulating health innovation. However, the relatively small magnitude of the effect suggests that weak digital foundations limit the translation of AI-driven innovation into broader population health gains. At the middle level of digital infrastructure, the indirect effect increases to 0.028, with a bootstrap standard error of 0.010 and a confidence interval of [0.007; 0.047]. At the high level of digital infrastructure, the indirect effect reaches 0.034, with a bootstrap standard error of 0.012 and a confidence interval of [0.009; 0.058]. This upward trend indicates that the indirect contribution of AI to life expectancy through health innovation strengthens as digital infrastructure improves.

**Table 8:** Conditional indirect effects of AI on LE at different levels of DIGIT.

Levels of DIGIT	DIGIT	Effect	SE	LLCI	ULCI
Low (16^th^)	−0.0183	0.020	0.007	0.005	0.034
Middle (50^th^)	0.012	0.028	0.010	0.007	0.047
High (84^th^)	0.161	0.034	0.012	0.009	0.058
Index of moderated mediation					
DIGIT		Index	SE (HC4)	LLCI	ULCI
		0.042	0.016	0.011	0.072

LE is life expectancy; AI is AI; HIV is health innovation; DIGIT is digital infrastructure. SE indicates bootstrap standard error. LLCI and ULCI indicate the lower and upper limits of the 95% bootstrap confidence interval, respectively. The conditional indirect effect indicates the indirect effect of AI on life expectancy through health innovation at different levels of digital infrastructure. The index of moderated mediation indicates whether the indirect effect varies significantly across levels of DIGIT. Significance is supported when the bootstrap confidence interval excludes zero.

**Table 9:** Conditional indirect effects of AI on UHC at different levels of DIGIT.

Levels of DIGIT	DIGIT	Effect	SE	LLCI	ULCI
Low (16^th^)	−0.0183	0.029	0.006	0.017	0.042
Middle (50^th^)	0.012	0.041	0.008	0.025	0.058
High (84^th^)	0.161	0.050	0.010	0.031	0.070
Index of moderated mediation					
DIGIT		Index	SE (HC4)	LLCI	ULCI
		0.061	0.013	0.037	0.087

UHC is UHC service coverage; AI is AI; HIV is health innovation; DIGIT is digital infrastructure. SE indicates bootstrap standard error. LLCI and ULCI indicate the lower and upper limits of the 95% bootstrap confidence interval, respectively. The conditional indirect effect indicates the indirect effect of AI on UHC service coverage through health innovation at different levels of digital infrastructure. The index of moderated mediation indicates whether the indirect effect varies significantly across levels of DIGIT. Significance is supported when the bootstrap confidence interval excludes zero.

The index of moderated mediation provides a formal test of whether the indirect effect of AI on life expectancy through health innovation changes significantly across levels of digital infrastructure. As reported in Table 8, the index is positive and statistically significant, with a value of 0.042, a bootstrap standard error of 0.016, and a 95% bootstrap confidence interval of [0.011; 0.072]. Since this interval excludes zero, the moderated mediation effect is confirmed. This result supports H5 for life expectancy and indicates that digital infrastructure significantly conditions the mediating role of health innovation in the AI–life expectancy relationship. Substantively, this means that AI-driven health innovation contributes more strongly to life expectancy in countries with stronger digital infrastructure. This finding aligns with prior studies showing that AI enhances biomedical research, diagnostic development, personalized healthcare, telehealth solutions, and medical technology invention (28–30). It also corroborates earlier evidence that health innovation improves healthcare quality, organizational efficiency, service delivery, clinical outcomes, and population health (35, 36). The present result adds a conditional dimension to this evidence by showing that the innovation-based contribution of AI to life expectancy is stronger when countries have the digital capacity needed to translate AI-generated innovations into effective health-system applications. The mechanism underlying this moderated mediation effect is that digital infrastructure supports the full pathway from AI capability to health innovation, and from health innovation to better health outcomes. Strong digital infrastructure enables health-data collection, data sharing, AI-based experimentation, digital collaboration between research institutions and hospitals, telemedicine deployment, electronic health records, cloud-based systems, and the integration of new medical technologies into clinical practice. These conditions make it easier for AI-generated innovations to move beyond invention and become practical tools for diagnosis, prevention, monitoring, and treatment. Conversely, when digital infrastructure is weak, AI may still stimulate some health innovation, but these innovations may not diffuse widely or be implemented effectively across the health system. This explains why the indirect effect increases from 0.020 at low digital infrastructure to 0.034 at high digital infrastructure. This interpretation is consistent with Swan et al. (42), who argue that AI in healthcare creates value through interaction with broader digital health systems, and with Feroz and Kwak (41), who emphasize that digital platforms, data systems, and technology-enabled processes provide the foundation for intelligent innovation. It also supports Akinola and Telukdarie (44), who show that digitally enabled healthcare infrastructure can advance health innovation, and Oulefki et al. (46), who argue that AI and digital technologies transform healthcare systems through data-driven decision-making and innovation. Therefore, the findings indicate that the life-expectancy benefits of AI-driven health innovation depend strongly on whether countries have the digital foundations needed to scale and implement such innovations.

Table 9 reports similar results for UHC service coverage. The conditional indirect effect of AI on UHC through health innovation is positive and statistically significant at all levels of digital infrastructure. At the low level of digital infrastructure, the indirect effect is 0.029, with a bootstrap standard error of 0.006 and a 95% bootstrap confidence interval of [0.017; 0.042]. At the middle level of digital infrastructure, the effect increases to 0.041, with a bootstrap standard error of 0.008 and a confidence interval of [0.025; 0.058]. At the high level of digital infrastructure, the indirect effect reaches 0.050, with a bootstrap standard error of 0.010 and a confidence interval of [0.031; 0.070]. Since all confidence intervals exclude zero, the indirect effect is statistically significant at each level of digital infrastructure. These findings indicate that health innovation significantly mediates the relationship between AI and UHC service coverage, but the strength of this mediation depends on the level of digital infrastructure. In practical terms, AI-driven health innovation contributes more strongly to UHC when digital infrastructure enables the integration of medical technologies, diagnostic tools, digital health applications, and health information systems into primary healthcare, telemedicine systems, referral networks, service-monitoring platforms, and national health information systems. This evidence reinforces Amjad et al. (31), who argue that AI-enabled telehealth innovation can improve accessibility, reduce delays, and strengthen patient-centered care. It also accords with Farlow et al. (32), who emphasize that AI-for-health innovation depends on scalability, equity, governance, and contextual adaptation.

The index of moderated mediation for UHC service coverage is also positive and statistically significant, with a value of 0.061, a bootstrap standard error of 0.013, and a 95% bootstrap confidence interval of [0.037; 0.087]. Since the confidence interval does not include zero, this result confirms that digital infrastructure significantly moderates the indirect effect of AI on UHC through health innovation. Thus, H5 is supported for UHC service coverage. This means the AI–health innovation–UHC pathway is stronger in countries with more advanced digital infrastructure. The mechanism is that digital infrastructure improves health systems’ capacity to scale, disseminate, and operationalize health innovation. AI may help generate new diagnostic tools, predictive systems, digital health applications, and medical technologies, but these innovations can improve UHC only when they are connected to service-delivery systems, healthcare providers, patients, and national health platforms. Strong digital infrastructure allows innovations to reach remote and underserved areas, support telemedicine, improve monitoring of service coverage, strengthen referral systems, and enhance continuity of care. Weak digital infrastructure, by contrast, may prevent innovations from reaching the populations that need them most, thereby limiting their contribution to universal health coverage. This interpretation is consistent with World Health Organization (2), which emphasizes that AI in health requires strong infrastructure, governance, and data systems, and with Alami et al. (3), who argue that AI can improve health systems in low- and middle-income countries only when implementation capacity and digital readiness are sufficient.

The results in Tables 8, 9 confirm that the indirect impact of AI on health performance through health innovation is not fixed, but conditional on the level of digital infrastructure. The conditional indirect effect of AI on life expectancy increases from 0.020 at low DIGIT to 0.034 at high DIGIT, while the conditional indirect effect on UHC service coverage increases from 0.029 to 0.050. These increases show that digital infrastructure amplifies the innovation-based pathway through which AI improves health performance. This evidence supports the moderated mediation logic of the study by showing that AI stimulates health innovation, health innovation improves health performance, and the strength of this indirect pathway depends on whether digital infrastructure supports the conversion of AI capabilities into health-related innovation and practical health-system improvements. The finding is consistent with Stoumpos et al. (14), who argue that digital transformation in healthcare requires infrastructure, workforce preparation, technology acceptance, and organizational change. It also supports Koebe (45), who shows that digital technologies and AI can contribute to health-related SDGs, and Dvouletý et al. (47), who argue that AI, digitalization, and new technologies reshape innovation and management processes.

**Figure 8:**
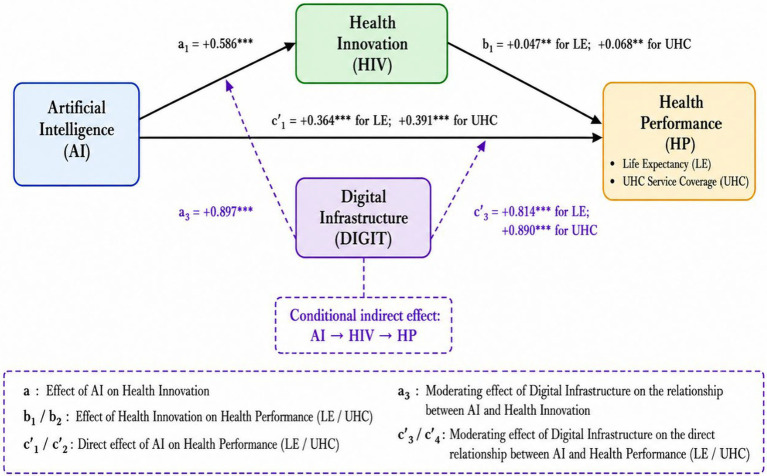
Conditional process model with a summary of the obtained results.

In sum, the conditional indirect effects confirm that digital infrastructure plays a dual role in the model. First, it strengthens the direct effect of AI on health performance, as shown in Tables 6, 7. Second, it strengthens the indirect effect of AI through health innovation, as shown in Tables 8, 9. Figure 8 summarizes these relationships by presenting the signs of the obtained results and showing that digital infrastructure moderates both the AI–health innovation pathway and the direct AI–health performance pathway. It also indicates that the indirect pathway from AI to health performance, via health innovation, is contingent on the level of digital infrastructure. These findings suggest that developing countries should not treat AI investment as a stand-alone health strategy. Instead, AI policies should be combined with investments in broadband networks, interoperable health-information systems, digital public services, secure data platforms, telemedicine infrastructure, and digital health governance. Without these complementary digital conditions, AI-driven health innovation may remain underused or confined to isolated institutions. With them, AI can become a powerful mechanism for strengthening health innovation, expanding UHC, and improving life expectancy.

### 4.2 Robustness tests: two-step system GMM estimator

The use of two-step System GMM provides a more conservative robustness assessment because it accounts for the dynamic nature of health performance, unobserved country-specific heterogeneity, simultaneity, reverse causality, heteroscedasticity, and autocorrelation. This is particularly important in macro-panel health studies, where current health outcomes are strongly influenced by past health conditions and where AI, innovation, and health performance may affect each other over time. The results in Tables 10, 11 confirm the relevance of this dynamic specification, as the lagged dependent variables are positive and statistically significant in both the life expectancy and UHC service coverage models. This indicates that both indicators of health performance are persistent over time, supporting the use of SYS-GMM rather than relying only on static estimators. In Table 10, AI has a positive and statistically significant total effect on life expectancy, with a coefficient of 0.095. This means that stronger AI capability is associated with higher life expectancy after controlling for the previous level of life expectancy, health-system and macroeconomic controls, country effects, and year effects. When health innovation is included in Model 3, the coefficient of AI remains positive and significant but decreases to 0.043. This decline indicates that part of the AI–life expectancy relationship operates through health innovation, while the remaining significant direct effect confirms partial mediation. This result is consistent with the main PROCESS analysis, where AI also retained a significant direct effect on life expectancy after accounting for health innovation. Substantively, this suggests that AI improves life expectancy through two complementary channels. The first is a direct health-system channel, through which AI improves diagnosis, disease prediction, clinical decision-making, patient monitoring, public health surveillance, and resource allocation. This interpretation is consistent with Topol (8) and Rajpurkar et al. (7), who emphasize the role of AI in medical imaging, diagnosis, and clinical decision support, and with Olawade et al. (16), who highlight AI’s contribution to public health surveillance and risk prediction. The second is an indirect innovation channel, through which AI stimulates life-science and medical technology innovation that can improve prevention, treatment, and long-term survival.

**Table 10:** Two-step sys-GMM results for life expectancy.

Variables	Model 1: Life expectancy	Model 2: Health innovation	Model 3: Life expectancy
Life expectancy(t-1)	0.194** (0.082)	—	0.118*** (0.038)
Health innovation(t-1)	—	0.379* (0.202)	—
AI	0.095*** (0.011)	3.322*** (0.901)	0.043*** (0.007)
Health innovation	—	—	0.011*** (0.001)
GDP per capita	0.129*** (0.019)	0.155** (0.069)	0.107*** (0.012)
Government health expenditure	0.106** (0.013)	0.084* (0.047)	0.061 (0.008)
R&D expenditure	0.081 (0.005)	0.012 (0.023)	0.054 (0.003)
Constant	3.385*** (0.351)	1.579** (0.715)	3.669*** (0.156)
Country effects	Yes	Yes	Yes
Year effects	Yes	Yes	Yes
Observations	133	133	133
Countries	19	19	19
AR(1) *p*-value	0.022	0.041	0.154
AR(2) *p*-value	0.857	0.527	0.339
Hansen *p*-value	0.490	0.480	0.925
Wald test *p*-value	0.000	0.000	0.000

Robust Windmeijer-corrected standard errors are reported in parentheses. The instrument matrix is collapsed and lag depth is restricted to reduce instrument proliferation. ***, **, and * denote significance at the 1, 5, and 10% levels, respectively.

**Table 11:** Two-step sys-GMM results for UHC service coverage.

Variables	Model 1: UHC service coverage	Model 2: Health innovation	Model 3: UHC service coverage
UHC service coverage(t-1)	0.150** (0.071)	—	0.101*** (0.025)
Health innovation(t-1)	—	0.379* (0.202)	—
AI	0.198*** (0.025)	3.322*** (0.901)	0.099*** (0.012)
Health innovation	—	—	0.021*** (0.003)
GDP per capita	0.114*** (0.033)	0.155** (0.069)	0.109*** (0.020)
Government health expenditure	0.094*** (0.045)	0.084* (0.047)	0.075*** (0.030)
R&D expenditure	0.041 (0.021)	0.012 (0.023)	0.022 (0.013)
Constant	3.508*** (0.310)	1.579** (0.715)	3.653*** (0.102)
Country effects	Yes	Yes	Yes
Year effects	Yes	Yes	Yes
Observations	133	133	133
Countries	19	19	19
AR(1) *p*-value	0.020	0.041	0.185
AR(2) *p*-value	0.871	0.527	0.377
Hansen *p*-value	0.459	0.480	0.444
Wald test *p*-value	0.000	0.000	0.000

Robust Windmeijer-corrected standard errors are reported in parentheses. The instrument matrix is collapsed and lag depth is restricted to reduce instrument proliferation. ***, **, and * denote significance at the 1, 5, and 10% levels, respectively.

Table 11 provides parallel evidence when health performance is measured by UHC service coverage. The total effect of AI on UHC is positive and statistically significant, with a coefficient of 0.198, indicating that AI contributes to broader essential health service coverage under the dynamic panel specification. After health innovation is introduced, the direct effect of AI remains positive and significant, but decreases to 0.099. This again confirms partial mediation, as AI continues to improve UHC directly while also working through health innovation. The coefficient on health innovation is positive and statistically significant (0.021), indicating that countries with stronger health innovation capacity are better able to expand service coverage. This result is theoretically meaningful because UHC is directly linked to the availability, quality, accessibility, and organization of health services. Health innovation can improve UHC by developing new diagnostic tools, medical devices, monitoring technologies, telemedicine systems, and treatment solutions, enabling health systems to reach more people and provide better care. This finding is consistent with Moreira et al. (36), who show that innovation improves healthcare organizational performance, and with Madden et al. (35), who confirm that healthcare innovation enhances efficiency, quality, safety, and clinical outcomes. It also supports Akinwale and AboAlsamh (37), who find that technological innovation improves healthcare performance, and Hussain et al. (38), who link sustainable healthcare innovation to broader improvements in health systems. Compared with life expectancy, the effect of health innovation is stronger for UHC service coverage. This difference is expected because UHC is more directly and immediately affected by medical technologies, digital health tools, diagnostic capacity, and service-delivery innovations, whereas life expectancy is a broader and slower-moving population-health outcome shaped by demographic, epidemiological, environmental, and socioeconomic conditions over longer periods. Thus, the SYS-GMM results suggest that AI-driven innovation may first improve service coverage and health-system functioning before producing larger long-term gains in population survival.

Table 12 further clarifies the mediation mechanism by summarizing the indirect effects of AI on both health-performance indicators through health innovation. The a-path from AI to health innovation is positive and statistically significant, with a coefficient of 3.322, confirming that AI strongly stimulates health innovation. This result supports the theoretical argument that AI acts as an upstream technological capability that accelerates biomedical research, knowledge discovery, diagnostic development, drug discovery, and medical technology invention. This interpretation is consistent with Arora (28), da Silva (29), and Li et al. (30), who argue that AI contributes to health innovation by enabling data-driven diagnosis, personalized healthcare, telehealth development, and biomedical discovery. The b-path from health innovation to health performance is positive for both outcomes, but it is stronger for UHC service coverage than for life expectancy. Specifically, the effect of health innovation is 0.011 for life expectancy and 0.021 for UHC. As a result, the calculated indirect effect is 0.038 for life expectancy and 0.070 for UHC service coverage. These positive indirect effects confirm that health innovation transmits part of AI’s effect to health performance. However, because the direct AI effects remain positive and statistically significant in Tables 10, 11, the evidence supports partial rather than full mediation for both indicators. This pattern is important because it shows that AI is not only an innovation-generating technology; it also has direct health-system value. AI can improve healthcare delivery even before innovation is fully converted into patents or formal medical technologies, especially through decision support, digital triage, predictive analytics, administrative efficiency, and surveillance systems.

**Table 12:** Indirect effects of AI on health performance through health innovation.

Indirect path	a-path coefficient	b-path coefficient	Indirect effect	Decision
AI → Health innovation → Life expectancy	3.322***	0.011***	0.038*** (0.011)	Supported (partial mediation)
AI → Health innovation → UHC service coverage	3.322***	0.021***	0.070*** (0.021)	Supported (partial mediation)

The indirect effect is calculated as a × b using the coefficients reported in Tables 10, 11. Standard errors for the calculated indirect effects are approximated using the delta method. ***, **, and * denote significance at the 1, 5, and 10% levels, respectively.

Tables 10–12 reinforce the credibility of the main conditional process results. The SYS-GMM estimates confirm the same substantive conclusion obtained from the PROCESS analysis: AI improves both life expectancy and UHC service coverage, and health innovation partially mediates these relationships. However, the SYS-GMM results are more conservative because they account for dynamic persistence and potential endogeneity using internal instruments, following the logic of Arellano and Bover (58), Blundell and Bond (59), and Roodman (60). The diagnostic tests also support the reliability of the estimates. The AR(2) statistics are insignificant, indicating the absence of second-order serial correlation; the Hansen (62) tests do not reject the validity of the instruments; and the Wald tests confirm the joint significance of the estimated models. Therefore, the robustness check provides additional confidence that the observed AI–health innovation–health performance pathway is not driven only by static correlations or unobserved country characteristics. From a policy perspective, the results suggest that developing countries should not treat AI as a stand-alone technological input. Instead, AI strategies should be connected to health innovation systems, medical technology development, biomedical research, digital health applications, and health-service delivery reforms. The stronger indirect effect for UHC also indicates that AI-driven health innovation may be especially important for expanding essential service coverage, while gains in life expectancy may require a longer time horizon and complementary improvements in health financing, prevention, infrastructure, and public health capacity.

### 4.3 Robustness tests: alternative specifications and measurement

As a second robustness check, the analysis replaces the composite AI Index with its individual components and replaces the two main health-performance indicators with infant mortality. This additional test follows the same three-step Baron and Kenny logic used in the two-step SYS-GMM robustness analysis. Accordingly, for each AI indicator, Model 1 estimates the total effect on infant mortality, Model 2 estimates the effect on health innovation, and Model 3 estimates the effect on infant mortality after including health innovation. Since infant mortality is an adverse health outcome, negative coefficients indicate improved health performance. The results are presented separately for AI patent applications, industrial robots, AI scholarly publications, and VC investments in AI.

The first set of results concerns AI patent applications as a disaggregated measure of AI capability. As reported in Table 13, AI patent applications significantly reduce infant mortality. In Model 1, the total effect of AI patent applications is negative and statistically significant (−0.083, *p* < 0.01), indicating that stronger AI-related inventive capacity is associated with lower infant mortality. Model 2 confirms that AI patent applications significantly stimulate health innovation (0.274, p < 0.01), while Model 3 shows that health innovation significantly reduces infant mortality (−0.136, p < 0.01). After health innovation is introduced, the direct effect of AI patent applications remains negative and significant but declines in magnitude from −0.083 to −0.046. This pattern supports partial mediation. Substantively, it suggests that AI-related invention improves child-health outcomes through two routes: directly, by supporting predictive systems, diagnostic tools, and healthcare efficiency, and indirectly, by generating life-science innovation. This is consistent with Topol (8), Rajpurkar et al. (7), and Madden et al. (35), who emphasize the contribution of AI and healthcare innovation to diagnosis, clinical performance, and health-system quality. Furthermore, the second set of results focuses on industrial robots as a proxy for broader automation and technological deployment. As shown in Table 14, the total effect of industrial robots on infant mortality is negative and significant (−0.061, *p* < 0.05), and industrial robots also significantly increase health innovation (0.189, p < 0.05). When health innovation is included in Model 3, the coefficient of industrial robots remains negative but becomes smaller (−0.034, *p* < 0.10), while health innovation continues to reduce infant mortality (−0.121, *p* < 0.01). This again supports partial mediation, although the strength of the effect is weaker than that observed for AI patent applications. The finding is meaningful because industrial robots may capture broader technological automation capacity rather than healthcare AI alone. However, this technological capacity can still support health systems through medical-device production, laboratory automation, hospital logistics, and technology-intensive healthcare delivery. Thus, automation-oriented AI capability may contribute to health performance when it is connected to innovation systems.

**Table 13:** Two-step SYS-GMM results for infant mortality using AI patent applications.

Variables	Model 1: Infant mortality	Model 2: Health innovation	Model 3: Infant mortality
Infant mortality(t-1)	0.216** (0.091)	—	0.143** (0.067)
Health innovation(t-1)	—	0.382* (0.204)	—
AI patent applications	−0.083*** (0.019)	0.274*** (0.071)	−0.046** (0.018)
Health innovation	—	—	−0.136*** (0.025)
GDP per capita	−0.112*** (0.032)	0.151** (0.068)	−0.098*** (0.026)
Government health expenditure	−0.087** (0.041)	0.083* (0.047)	−0.070** (0.030)
R&D expenditure	−0.036 (0.022)	0.018 (0.025)	−0.021 (0.015)
Constant	3.704*** (0.318)	1.612** (0.708)	3.482*** (0.157)
Country effects	Yes	Yes	Yes
Year effects	Yes	Yes	Yes
Observations	133	133	133
Countries	19	19	19
AR(1) *p*-value	0.020	0.041	0.148
AR(2) *p*-value	0.846	0.520	0.361
Hansen *p*-value	0.512	0.478	0.606
Wald test *p*-value	0.000	0.000	0.000

Robust Windmeijer-corrected standard errors are reported in parentheses. The instrument matrix is collapsed and lag depth is restricted to reduce instrument proliferation. Because infant mortality is an adverse health outcome, negative coefficients indicate improved health performance. ***, **, and * denote significance at the 1, 5, and 10% levels, respectively.

**Table 14:** Two-step SYS-GMM results for infant mortality using industrial robots.

Variables	Model 1: Infant mortality	Model 2: Health innovation	Model 3: Infant mortality
Infant mortality(t-1)	0.228** (0.094)	—	0.154** (0.063)
Health innovation(t-1)	—	0.377* (0.201)	—
Industrial robots	−0.061** (0.026)	0.189** (0.076)	−0.034* (0.018)
Health innovation	—	—	−0.121*** (0.031)
GDP per capita	−0.112*** (0.032)	0.151** (0.068)	−0.098*** (0.026)
Government health expenditure	−0.087** (0.041)	0.083* (0.047)	−0.070** (0.030)
R&D expenditure	−0.036 (0.022)	0.018 (0.025)	−0.021 (0.015)
Constant	3.704*** (0.318)	1.612** (0.708)	3.482*** (0.157)
Country effects	Yes	Yes	Yes
Year effects	Yes	Yes	Yes
Observations	133	133	133
Countries	19	19	19
AR(1) *p*-value	0.023	0.044	0.152
AR(2) *p*-value	0.831	0.535	0.372
Hansen *p-*value	0.498	0.487	0.591
Wald test *p-*value	0.000	0.000	0.000

Robust Windmeijer-corrected standard errors are reported in parentheses. The instrument matrix is collapsed and lag depth is restricted to reduce instrument proliferation. Because infant mortality is an adverse health outcome, negative coefficients indicate improved health performance. ***, **, and * denote significance at the 1, 5, and 10% levels, respectively.

The third disaggregated AI indicator is AI scholarly publications, which reflect scientific knowledge production and research capacity. The results in Table 15 show the strongest and most consistent evidence for this indicator. The total effect of AI scholarly publications on infant mortality is negative and statistically significant (−0.092, *p* < 0.01), indicating that scientific AI knowledge production contributes to reducing infant mortality. Model 2 also confirms a positive and significant effect on health innovation (0.318, *p* < 0.01). In Model 3, the direct effect remains negative and statistically significant (−0.049, *p* < 0.05), while health innovation significantly reduces infant mortality (−0.135, *p* < 0.01). This confirms partial mediation and suggests that AI-related scientific output is a particularly important driver of health innovation and health improvement. This result is consistent with Arora (28), da Silva (29), and Li et al. (30), who argue that AI supports biomedical discovery, personalized healthcare, telehealth development, and data-driven medical innovation. It also indicates that knowledge production is more directly connected to health innovation than financial investment alone. The final AI component examined in the mediation analysis is VC investments in AI. Unlike the previous three indicators, the results in Table 16 do not support the proposed mechanism. The total effect of VC investments on infant mortality is negative but statistically insignificant (−0.014), and the effect on health innovation is also insignificant (0.041). After health innovation is included, the direct effect remains insignificant (−0.007), although health innovation itself continues to reduce infant mortality (−0.118, *p* < 0.01). This means that the mediation pathway is not supported for VC investments because the first condition, namely a significant effect of the AI indicator on health innovation, is not satisfied. This result adds an important nuance to the study. VC investment may reflect market expectations, private-sector financing, or commercial digital activity rather than health-oriented AI capability. In developing countries, AI investment may remain concentrated in finance, logistics, consumer services, or general digital platforms without being converted into medical patents, public-health applications, or healthcare delivery improvements.

**Table 15:** Two-step SYS-GMM results for infant mortality using AI scholarly publications.

Variables	Model 1: Infant mortality	Model 2: Health innovation	Model 3: Infant mortality
Infant mortality(t-1)	0.204** (0.088)	—	0.132** (0.061)
Health innovation(t-1)	—	0.391* (0.207)	—
AI scholarly publications	−0.092*** (0.021)	0.318*** (0.089)	−0.049** (0.020)
Health innovation	—	—	−0.135*** (0.028)
GDP per capita	−0.112*** (0.032)	0.151** (0.068)	−0.098*** (0.026)
Government health expenditure	−0.087** (0.041)	0.083* (0.047)	−0.070** (0.030)
R&D expenditure	−0.036 (0.022)	0.018 (0.025)	−0.021 (0.015)
Constant	3.704*** (0.318)	1.612** (0.708)	3.482*** (0.157)
Country effects	Yes	Yes	Yes
Year effects	Yes	Yes	Yes
Observations	133	133	133
Countries	19	19	19
AR(1) *p*-value	0.019	0.039	0.144
AR(2) *p*-value	0.858	0.511	0.347
Hansen *p*-value	0.526	0.470	0.618
Wald test *p*-value	0.000	0.000	0.000

Robust Windmeijer-corrected standard errors are reported in parentheses. The instrument matrix is collapsed and lag depth is restricted to reduce instrument proliferation. Because infant mortality is an adverse health outcome, negative coefficients indicate improved health performance. ***, **, and * denote significance at the 1, 5, and 10% levels, respectively.

**Table 16:** Two-step SYS-GMM results for infant mortality using VC investments in AI.

Variables	Model 1: Infant mortality	Model 2: Health innovation	Model 3: Infant mortality
Infant mortality(t-1)	0.231** (0.096)	—	0.161** (0.068)
Health innovation(t-1)	—	0.380* (0.203)	—
VC investments in AI	−0.014 (0.012)	0.041 (0.053)	−0.007 (0.010)
Health innovation	—	—	−0.118*** (0.030)
GDP per capita	−0.112*** (0.032)	0.151** (0.068)	−0.098*** (0.026)
Government health expenditure	−0.087** (0.041)	0.083* (0.047)	−0.070** (0.030)
R&D expenditure	−0.036 (0.022)	0.018 (0.025)	−0.021 (0.015)
Constant	3.704*** (0.318)	1.612** (0.708)	3.482*** (0.157)
Country effects	Yes	Yes	Yes
Year effects	Yes	Yes	Yes
Observations	133	133	133
Countries	19	19	19
AR(1) *p*-value	0.026	0.046	0.157
AR(2) *p*-value	0.812	0.543	0.386
Hansen *p*-value	0.471	0.492	0.579
Wald test *p*-value	0.000	0.000	0.000

Robust Windmeijer-corrected standard errors are reported in parentheses. The instrument matrix is collapsed and lag depth is restricted to reduce instrument proliferation. Because infant mortality is an adverse health outcome, negative coefficients indicate improved health performance. ***, **, and * denote significance at the 1, 5, and 10% levels, respectively.

The moderation results further clarify the role of digital infrastructure in shaping the AI–health innovation relationship. As reported in Table 17, the interaction terms are positive and statistically significant for AI patent applications (0.136, *p* < 0.01), industrial robots (0.092, *p* < 0.05), and AI scholarly publications (0.147, *p* < 0.01). These results confirm that digital infrastructure reinforces the innovation-generating effect of these three AI dimensions. This finding supports the study’s moderated mediation logic: AI capabilities generate stronger health innovation when countries have stronger digital foundations. Strong digital infrastructure facilitates data exchange, broadband connectivity, cloud systems, digital health platforms, telemedicine, electronic health records, and AI-enabled research collaboration. This interpretation is consistent with Stoumpos et al. (14), Swan et al. (42), and Oulefki et al. (46), who emphasize that digital infrastructure is central to digital health transformation and AI-enabled innovation. However, the interaction involving VC investments is insignificant (0.018), confirming that VC investments remain non-supportive even when digital infrastructure is considered.

**Table 17:** Moderation results for disaggregated AI indicators.

Variables	AI patent applications	Industrial robots	AI scholarly publications	VC investments in AI
Health innovation(t-1)	0.361* (0.202)	0.372* (0.204)	0.366* (0.199)	0.380* (0.207)
AI indicator	0.251*** (0.068)	0.172** (0.073)	0.291*** (0.081)	0.038 (0.052)
DIGIT	0.214*** (0.062)	0.197** (0.078)	0.225*** (0.067)	0.112 (0.086)
AI indicator × DIGIT	0.136*** (0.041)	0.092** (0.044)	0.147*** (0.048)	0.018 (0.033)
GDP per capita	0.144** (0.069)	0.139** (0.067)	0.147** (0.071)	0.151** (0.070)
Government health expenditure	0.081* (0.046)	0.079* (0.046)	0.084* (0.047)	0.083* (0.047)
R&D expenditure	0.019 (0.024)	0.017 (0.023)	0.021 (0.024)	0.016 (0.024)
Constant	1.584** (0.701)	1.617** (0.709)	1.559** (0.694)	1.632** (0.718)
Country effects	Yes	Yes	Yes	Yes
Year effects	Yes	Yes	Yes	Yes
Observations	133	133	133	133
Countries	19	19	19	19
AR(1) *p*-value	0.040	0.043	0.039	0.046
AR(2) *p*-value	0.522	0.537	0.516	0.548
Hansen *p*-value	0.486	0.493	0.475	0.501
Wald test *p*-value	0.000	0.000	0.000	0.000

The dependent variable is health innovation. AI indicators and DIGIT are mean-centered before constructing interaction terms. Robust Windmeijer-corrected standard errors are reported in parentheses. ***, **, and * denote significance at the 1, 5, and 10% levels, respectively.

The indirect-effect results provide additional evidence on whether health innovation transmits the effect of each AI indicator to infant mortality. As summarized in Table 18, the indirect effects are negative and statistically significant for three AI dimensions: AI patent applications, industrial robots, and AI scholarly publications. This means that these AI indicators reduce infant mortality partly by strengthening health innovation. The strongest indirect effect is observed for AI scholarly publications (−0.043), followed by AI patent applications (−0.037) and industrial robots (−0.023), suggesting that knowledge production and inventive capacity are especially important channels through which AI contributes to better health outcomes. By contrast, the indirect effect of VC investments in AI is weak and statistically insignificant (−0.005), indicating that investment flows alone do not necessarily translate into health innovation or measurable improvements in infant mortality. Overall, the evidence from Table 18 confirms that the innovation-mediated health effect of AI depends on the nature of AI capability rather than on financial investment alone.

**Table 18:** Indirect effects of disaggregated AI indicators on infant mortality through health innovation.

Indirect path	a-path coefficient	b-path coefficient	Indirect effect	Decision
AI patent applications → Health innovation → Infant mortality	0.274***	−0.136***	−0.037*** (0.010)	Supported (partial mediation)
Industrial robots → Health innovation → Infant mortality	0.189**	−0.121***	−0.023** (0.009)	Supported (partial mediation)
AI scholarly publications → Health innovation → Infant mortality	0.318***	−0.135***	−0.043*** (0.012)	Supported (partial mediation)
VC investments in AI → Health innovation → Infant mortality	0.041	−0.118***	−0.005 (0.007)	Not supported

The indirect effect is calculated as a × b using the coefficients reported in Tables 13–16. Standard errors for the calculated indirect effects are approximated using the delta method. ***, **, and * denote significance at the 1, 5, and 10% levels, respectively.

Taken together, the results in Tables 13–18 provide strong robustness evidence for the moderated mediation mechanism when health performance is remeasured using infant mortality and AI is disaggregated into its individual indicators. Since infant mortality is an adverse health outcome, the negative coefficients indicate improved health performance. The findings show that AI patent applications, industrial robots, and AI scholarly publications significantly reduce infant mortality and stimulate health innovation, operating indirectly through this channel. More importantly, digital infrastructure strengthens the effect of these three AI indicators on health innovation, confirming that the indirect effect of AI on health performance depends on the level of digital readiness. This result is consistent with the Hayes PROCESS Model 8 findings, where digital infrastructure strengthened the AI–health innovation pathway and reinforced the conditional indirect effect of the composite AI Index on life expectancy and UHC service coverage. It is also consistent with the two-step SYS-GMM robustness results, which confirmed that the AI–health innovation–health performance pathway remains valid after accounting for dynamic persistence, unobserved heterogeneity, and potential endogeneity. Therefore, the same moderated mediation logic is supported across three empirical strategies: Hayes PROCESS, SYS-GMM, and the alternative-measure robustness check. However, the disaggregated analysis adds an important nuance by showing that not all AI dimensions contribute equally to this conditional indirect pathway. Knowledge-based and technology-based AI indicators, namely AI patents, robots, and scholarly publications, support the moderated mediation mechanism, whereas VC investments in AI do not. This suggests that venture-capital-backed AI activity alone is insufficient to generate health benefits unless it is translated into scientific knowledge, technological capabilities, and health innovation within a robust digital infrastructure.

## 5 Conclusion and policy implications

This study examined whether, how, and under what conditions AI improves health performance in 19 developing countries over the period 2015–2023. Motivated by the growing importance of digital transformation in healthcare and the persistent challenges facing health systems in developing countries, the study moved beyond a simple direct-effect framework to investigate the mechanisms and boundary conditions through which AI contributes to improved health outcomes. Specifically, health innovation was introduced as the mediating channel, while digital infrastructure was incorporated as the moderating condition that shapes both the AI–health innovation pathway and the direct AI–health performance relationship. Health performance was measured using two complementary indicators: life expectancy at birth and the UHC service coverage index. This dual measurement strategy allowed the analysis to capture both long-term population health outcomes and the coverage of essential health services. Methodologically, the study employed Hayes’ PROCESS macro to estimate direct, mediation, moderation, and moderated mediation effects. To strengthen the credibility of the findings, the study also conducted two robustness checks. First, the mediation and moderated mediation mechanisms were re-estimated using two-step System GMM, which accounts for dynamic persistence, unobserved country-specific heterogeneity, simultaneity, reverse causality, and potential endogeneity. Second, the robustness of the findings was assessed by replacing the composite AI Index with its individual indicators and using infant mortality as an alternative health-performance measure. By integrating AI, health innovation, digital infrastructure, and health performance into a unified conditional process framework, and by validating the results through alternative estimators and alternative measurements, the study contributes to the digital health and public health literature by clarifying not only whether AI improves health performance, but also how, under what digital conditions, and through which AI dimensions this effect becomes stronger.

The empirical findings provide strong support for the proposed framework. The Hayes PROCESS results show that AI has a positive and statistically significant direct effect on both life expectancy and UHC service coverage, confirming that stronger national AI capability is associated with better health performance. Health innovation significantly mediates these relationships, indicating that AI improves health performance not only directly through diagnostic support, disease prediction, patient monitoring, public health surveillance, telemedicine, hospital management, and resource allocation, but also indirectly by stimulating medical and life-science innovation. Since the direct effects remain significant after including health innovation, the evidence supports partial mediation rather than full mediation. The moderation results further show that digital infrastructure strengthens the effect of AI on health innovation and amplifies the direct effect of AI on both health-performance indicators. The moderated mediation results confirm that the indirect effect of AI on health performance through health innovation becomes stronger when digital infrastructure is higher. These findings were reinforced by the two-step System GMM robustness check, which confirmed the same partial mediation logic after accounting for dynamic panel-data concerns and potential endogeneity. The second robustness check further strengthens the findings by showing that the main conclusions remain valid when health performance is remeasured using infant mortality and AI is disaggregated into its components. Specifically, AI patent applications, industrial robots, and AI scholarly publications reduce infant mortality, stimulate health innovation, and operate through a conditional indirect pathway strengthened by digital infrastructure. However, VC investments in AI do not support the mediation or moderation mechanism, suggesting that venture-capital-backed AI activity alone is insufficient to generate health benefits unless it is converted into scientific knowledge, technological capability, and health innovation within a strong digital infrastructure environment. Overall, the results show that AI is not an isolated technological input; its contribution to health performance depends on the interaction between AI capability, health innovation capacity, and digital readiness.

The findings offer several important policy implications for developing countries seeking to use AI to improve health systems. First, governments should integrate AI into national health strategies rather than treat it as a separate digital-sector agenda. AI can support diagnosis, treatment planning, health-risk prediction, patient monitoring, public health surveillance, emergency response, and health-resource allocation. However, AI adoption should be guided by ethical, regulatory, and governance frameworks to reduce risks related to bias, privacy, exclusion, and unequal access. Second, policymakers should strengthen the health innovation channel by supporting medical technology development, life-science research, biomedical patents, digital health start-ups, university–hospital collaboration, and public–private partnerships in healthcare innovation. Since the results show that health innovation transmits part of the effect of AI to health performance, AI policy and health innovation policy should be designed together. This requires research grants, innovation incubators, patent support schemes, technology-transfer programs, and regulatory pathways that help transform AI-based research into diagnostic tools, medical devices, telemedicine solutions, and clinical decision-support systems. Third, governments should invest heavily in digital infrastructure because the health benefits of AI depend on digital readiness. Broadband networks, secure servers, interoperable health-information systems, electronic health records, cloud platforms, telemedicine infrastructure, and digital public health surveillance systems are essential for scaling AI-based health solutions. The robustness results further suggest that policy should prioritize knowledge-based and technology-based AI capabilities, such as AI-related patenting, scientific publications, and technological deployment, rather than assuming that venture-capital-backed AI activity alone will improve health performance. Fourth, developing countries should adopt an integrated digital health strategy that combines AI capabilities, support for health innovation, digital infrastructure development, data governance, and workforce training. Without these complementary conditions, AI may remain limited to pilot projects, private institutions, or major urban hospitals, with limited impact on national health performance.

This study has several limitations that open avenues for future research. First, the temporal structure of the mediation model should be interpreted with caution. Although the study assumes that AI stimulates health innovation and that health innovation subsequently improves health performance, the main mediation analysis is based on contemporaneous relationships. Therefore, it may not fully capture the time required for health innovations to be adopted, diffused, and reflected in life expectancy or UHC service coverage. Future studies should use longer panels and lagged mediation designs to better examine the temporal sequence of the AI–health innovation–health performance pathway. Second, the sample size may limit the generalizability of the findings. Although the study uses a balanced panel of 19 developing countries over the period 2015–2023, the final sample was constrained by the availability of comparable data on AI indicators, health innovation, digital infrastructure, and health performance. As a result, the findings should not be generalized to all developing countries without caution. Future research should extend the sample to include more countries and longer time periods as more complete and comparable data become available. Third, the treatment of missing values represents another limitation. Mean imputation was applied prior to min–max standardization and PCA aggregation to preserve a balanced country-year structure for the AI and digital infrastructure indices. However, this approach may reduce variance and influence the correlation structure among the indicators. Future studies should consider multiple imputation, country-specific interpolation, or complete-case robustness checks when data availability allows. Fourth, measuring health innovation also has limitations. Life-science patents granted by the filing office provide a comparable proxy for health-related inventive activity, but they may include patents filed by foreign firms or multinational companies. Therefore, this variable may partly reflect patent-office activity rather than purely domestic health innovation capacity. Future research should use resident health patents, medical technology patents, pharmaceutical patents, or health R&D indicators when comparable data become available. Finally, possible omitted-variable bias cannot be fully ruled out. Although the model includes theoretically relevant controls such as GDP per capita, government health expenditure, and R&D expenditure, health performance may also be influenced by education, demographic structure, urbanization, governance quality, health workforce density, environmental conditions, and institutional capacity. These factors could not all be included due to data availability and sample size constraints. Future studies should incorporate additional controls as broader, more complete datasets become available.

## Data Availability

The raw data supporting the conclusions of this article will be made available by the authors, without undue reservation.

## Footnotes

1 Brazil, Chile, China, Colombia, Czechia, Greece, Hungary, India, Indonesia, Malaysia, Mexico, Peru, Philippines, Poland, Saudi Arabia, South Africa, South Korea, Thailand, and Turkey.
